# The knocking down of the oncoprotein Golgi phosphoprotein 3 in T98G cells of glioblastoma multiforme disrupts cell migration by affecting focal adhesion dynamics in a focal adhesion kinase-dependent manner

**DOI:** 10.1371/journal.pone.0212321

**Published:** 2019-02-19

**Authors:** Cecilia Arriagada, Charlotte Luchsinger, Alexis E. González, Tomás Schwenke, Gloria Arriagada, Hugo Folch, Pamela Ehrenfeld, Patricia V. Burgos, Gonzalo A. Mardones

**Affiliations:** 1 Department of Physiology, School of Medicine, Universidad Austral de Chile, Valdivia, Chile; 2 Center for Interdisciplinary Studies of the Nervous System (CISNe), Universidad Austral de Chile, Valdivia, Chile; 3 Fundación Ciencia y Vida, Santiago, Chile; 4 Departmento de Ciencias Biologicas, Facultad de Ciencias de la Vida, Universidad Andres Bello, Viña del Mar, Chile; 5 Department of Immunology, School of Medicine, Universidad Austral de Chile, Valdivia, Chile; 6 Department of Anatomy, Histology and Pathology, School of Medicine, Universidad Austral de Chile, Valdivia, Chile; 7 Center for Cell Biology and Biomedicine, School of Science and Medicine, Universidad San Sebastián, Santiago, Chile; 8 Center for Aging and Regeneration (CARE), Facultad de Ciencias Biológicas, Pontificia Universidad Católica de Chile, Santiago, Chile; Seoul National University College of Pharmacy, REPUBLIC OF KOREA

## Abstract

Golgi phosphoprotein 3 (GOLPH3) is a conserved protein of the Golgi apparatus that in humans has been implicated in tumorigenesis. However, the precise function of GOLPH3 in malignant transformation is still unknown. Nevertheless, clinicopathological data shows that in more than a dozen kinds of cancer, including gliomas, GOLPH3 could be found overexpressed, which correlates with poor prognosis. Experimental data shows that overexpression of GOLPH3 leads to transformation of primary cells and to tumor growth enhancement. Conversely, the knocking down of GOLPH3 in GOLPH3-overexpressing tumor cells reduces tumorigenic features, such as cell proliferation and cell migration and invasion. The cumulative evidence indicate that GOLPH3 is an oncoprotein that promotes tumorigenicity by a mechanism that impact at different levels in different types of cells, including the sorting of Golgi glycosyltransferases, signaling pathways, and the actin cytoskeleton. How GOLPH3 connects mechanistically these processes has not been determined yet. Further studies are important to have a more complete understanding of the role of GOLPH3 as oncoprotein. Given the genetic diversity in cancer, a still outstanding aspect is how in this inherent heterogeneity GOLPH3 could possibly exert its oncogenic function. We have aimed to evaluate the contribution of GOLPH3 overexpression in the malignant phenotype of different types of tumor cells. Here, we analyzed the effect on cell migration that resulted from stable, RNAi-mediated knocking down of GOLPH3 in T98G cells of glioblastoma multiforme, a human glioma cell line with unique features. We found that the reduction of GOLPH3 levels produced dramatic changes in cell morphology, involving rearrangements of the actin cytoskeleton and reduction in the number and dynamics of focal adhesions. These effects correlated with decreased cell migration and invasion due to affected persistence and directionality of cell motility. Moreover, the knocking down of GOLPH3 also caused a reduction in autoactivation of focal adhesion kinase (FAK), a cytoplasmic tyrosine kinase that regulates focal adhesions. Our data support a model in which GOLPH3 in T98G cells promotes cell migration by stimulating the activity of FAK.

## Introduction

Golgi phosphoprotein 3 (GOLPH3) is a highly conserved protein identified in proteomics analyses of the Golgi apparatus [1, 2]. GOLPH3, also known in higher eukaryotes as GMx33α, GPP34 or MIDAS, as well as its orthologue Vps74 in *Saccharomyces cerevisiae*, is a peripheral membrane protein that also distributes in a large cytosolic pool [1]. GOLPH3 is a very dynamic protein enriched at the *trans*-Golgi network [1, 3], and its association to Golgi membranes, as well as of Vps74, seems to depend on its binding to phosphatidylinositol-4-phospate [4, 5]. Although almost two decades have passed since its discovery, a picture of the precise function of GOLPH3 has not emerged yet. The first function suggested for GOLPH3 came from a study showing that in *Saccharomyces cerevisiae* ablation of the gene *VPS74* disrupts the retention at the Golgi of a subset of glycosyltransferases, resulting in the production of hypoglycosylated proteins [6, 7]. Later, it was shown that in human cells the knocking down of GOLPH3 perturbs the localization of at least three glycosyltransferases, impairing normal *N*- and *O*-glycosylation of a set of proteins, suggesting a role of GOLPH3 in sorting at the Golgi [8–10]. Intriguingly, GOLPH3 seems to participate in other cellular processes, in and out of the Golgi. These functions include modulation of the mechanistic target of rapamycin (mTOR) signaling pathway [11], actin cytoskeleton-mediated maintenance of Golgi architecture [4], anterograde and retrograde protein trafficking [4, 11], cell motility and extracellular matrix remodeling [12], cell survival after DNA damage [13], regulation of cytokinesis [14], activation of the NF-κB pathway [15], induction of epithelial-mesenchymal transition [16], neurogenesis [17], modulation of telomere function [18], regulation of the JAK2-STAT3 pathway [19], and a curious role as modulator of mitochondrial activity [20–22]. The precise molecular mechanisms in which GOLPH3 is involved in all these processes are largely uncharacterized. Moreover, it is unknown whether the participation of GOLPH3 in these apparent dissimilar processes is related to different outcomes of a unique activity or to multiple activities of this protein.

Adding to the puzzling function of GOLPH3, a conspicuous observation identified this protein as an overexpressed product of a frequent amplification of the chromosomal region 5p13 found in several types of solid human tumors [11]. Subsequent experimental and clinicopathological data led to propose GOLPH3 as the first oncoprotein of the Golgi apparatus [11, 23–25]. Moreover, an increasing number of reports show a strong correlation between overexpression of GOLPH3 and poor cancer prognosis [26]. For this reason, GOLPH3 has been postulated as biomarker of tumor progression for a variety of cancer types, including breast cancer [27], oral tongue cancer [28], glioblastoma multiforme [29], prostate cancer [30], esophageal squamous cell carcinoma [31], gastric cancer [32], renal cell carcinoma [33], hepatocellular carcinoma [34], non-small cell lung cancer [35], pancreatic ductal adenocarcinoma [36], epithelial ovarian carcinoma [37], bladder cancer [38], colorectal cancer [39], and melanoma [40]. On the other hand, a remarkable early finding showed that in a set of tumor types the overexpression of GOLPH3 enhances the activity of the mTOR-signaling pathway [11]. This suggested possible explanations to the tumorigenic effect of GOLPH3, because deregulation of mTOR signaling is associated to tumor progression and cancer [41]. Accordingly, inhibition of mTOR by rapamycin in cells that overexpress GOLPH3 results in detrimental effects on cell survival, cell proliferation, tumor growth, and cell migration and invasion [11, 42–44]. However, it has not been elucidated how the level of GOLPH3 expression impacts the activity of the mTOR-signaling pathway, and whether this contributes in different types of cancer cells to the same mechanisms that lead to malignancy. This is in part due to the multiple functions mentioned above attributed to GOLPH3, and hence a well-defined molecular linkage is still missing [45]. Nevertheless, evidence from studies with different types of cells suggests that GOLPH3 overexpression stimulates cell migration and invasion through a mechanism that involves at least the following events: increase in *N*-glycan sialylation of the cell-extracellular matrix adhesion protein β1 integrin [10], stimulation of the mTOR signaling pathway [42], small GTPase RhoA-dependent actin cytoskeleton reorganization [12], overexpression of the gene expression regulator Y-box binding protein-1 (YB1) [42], Golgi reorientation and membrane trafficking toward the leading edge [46], and enhancement of extracellular matrix degradation by metalloproteinases [47, 48]. The clarification as to whether these set of events are connected to a single tumorigenic activity of GOLPH3, as well as if they are common features elicited by GOLPH3 overexpression, needs further investigation. In this regard, we are investigating possible differences that could arise from GOLPH3 overexpression in different types of cancer cells. We have found in the breast cancer cell lines MDA-MB-231 and MCF7 distinct biochemical pools of GOLPH3, which correlates with differences in some cell biological properties of this protein that could be related to the unique tumorigenic features of these cells [49]. Thus, to understand the different roles that the overexpression of GOLPH3 have in different cancer cells, we have also set to compare the effect of knocking down its expression. In the present report, we show the case of the outcome on cell motility resulted from knocking down the overexpression of GOLPH3 in the human glioblastoma multiforme cell line T98G. Our data indicate that the reduction of the levels of GOLPH3 in these cells affected their directional persistence of migration, by a mechanism that involves FAK-dependent, perturbed focal adhesion dynamics.

## Materials and methods

### Cell culture and generation of cell lines

T98G cells were obtained from the American Type Culture Collection (Manassas, VA), and were maintained in DMEM medium supplemented with 10% heat-inactivated fetal bovine serum (FBS), 100 U/ml penicillin, 100 μg/ml streptomycin (Life Technologies), and 5 μg/ml plasmocin (InvivoGen, San Diego, CA), in a humidified incubator with 5% CO_2_ at 37°C. We generated T98G cell lines stably expressing either of two shRNA to target GOLPH3 (shGOLPH3#1 and shGOLPH3#2), which were delivered by lentiviral particles. The shRNA vector pLKO.1 encoding the 3’-UTR sequence 5'-CCGGGCTTGCTTCAATCATGGTTATCTCGAGATAACCATGATTGAAGCAAGCTTTTTG-3' of human GOLPH3 (shGOLPH3#1) was obtained from Sigma-Aldrich. The shRNA vector pGFP-C-shLenti containing the coding DNA sequence 5'-GGTAATCTGTAAGTCAGATGCTCCAACAG-3' of human GOLPH3 (shGOLPH3#2) was obtained from Origen Technologies. The shRNA vector pLKO.1 encoding the sequence 5'-CGCTGAGTACTTCGAAATGTC-3' of firefly luciferase was used to generate a control, T98G cell line. Lentiviral particles were generated using a method that we have described elsewhere [50].

### Antibodies and cell reagents

We used the following mouse monoclonal antibodies: clone AC-74 to β-Actin (Sigma-Aldrich), clone B-5-1-2 to α-tubulin (Sigma-Aldrich), clone VIN-11-5 to vinculin (Sigma-Aldrich), and clone 35/GM130 to GM130 (BD Biosciences). We used the following rabbit monoclonal antibody: clone D20B1 to Phospho-Tyr-397 of FAK (Cell Signaling). We used rabbit polyclonal antibodies to the following proteins: GOLPH3 (Abcam, cat # ab98023), and FAK (Cell Signaling, cat # 3285). We used a homemade, mouse polyclonal antibody to human GOLPH3 that we generated as follows: Human, recombinant GOLPH3, prepared as described elsewhere [49], was used for mice immunization. Antibodies were subsequently affinity purified from mice sera using recombinant GOLPH3 immobilized on Affi-Gel 10 (Bio-Rad Laboratories), following the manufacturer's instructions. HRP–conjugated secondary antibodies were from Jackson ImmunoResearch. The following fluorochrome-conjugated antibodies were from Life Technologies: Alexa Fluor-488– or -594–conjugated donkey anti mouse IgG, and Alexa Fluor-488– or -647–conjugated donkey anti rabbit IgG. Primary antibodies were used at a dilution 1/200 to 1/2000. HRP–or Alexa Fluor–conjugated secondary antibodies were used at dilutions 1/1000 to 1/20000, depending on their reactivity. Nocodazole was from Calbiochem, and the FAK inhibitor Compound PF-562271 was from Laviana Corporation, and was a kind gift of V. Torres (Universidad de Chile). Puromycin dihydrochloride and a cocktail of protease inhibitors were from Sigma-Aldrich. The fluorescent nuclear stain 4’,6-diamidino-2-phenylindole (DAPI), and Tetramethylrhodamine B isothiocyanate-conjugated phalloidin (TRITC-phalloidin) were from Life Technologies.

### Immunoblotting and densitometry quantification

Preparation of protein extracts from cultured cells, SDS-PAGE, and immunoblotting were carried out using methods that we have described previously [49, 51]. The amount of immunoblot signal from images with unsaturated pixels was estimated using ImageJ software (version 1.47h; [52]). For each condition, protein bands were quantified from at least three independent experiments.

### Phase-contrast microscopy, fluorescence microscopy, and image analysis

For phase-contrast microscopy, cells grown in glass coverslips were fixed in 4% paraformaldehyde for 1 h at room temperature, and the coverslips were mounted onto glass slides using Fluoromount-G mounting medium (SouthernBiotech). Images were acquired with an AxioObserver.D1 microscope equipped with a LD A-Plan 20x objective (NA 0.3; Ph1) and an AxioCam MRm digital camera using AxioVision software (Carl Zeiss). For fluorescence microscopy, cells grown in glass coverslips were processed as we have described elsewhere [49]. For immunofluorescence, and depending on primary antibody reactivity, cells were fixed in 100% methanol or 4% paraformaldehyde. For TRITC-phalloidin decoration, cells were fixed only in 4% paraformaldehyde. Fluorescence microscopy images were acquired with an AxioObserver.D1 microscope equipped with a PlanApo 63x oil immersion objective (NA 1.4), and an AxioCam MRm digital camera, using AxioVision software (Carl Zeiss). Quantification of cell attachment area and the cell shape parameters aspect ratio and circularity index were performed with ImageJ software (version 1.47h; [53]), using the tool *Analyze/Measure* selecting in the dialog box *Set Measurements* the checkboxes *Area* and *Shape descriptors* as described elsewhere (https://imagej.nih.gov/ij/docs/guide/user-guide.pdf). To prepare figures, images were processed with ImageJ software or Adobe Photoshop CS3 software (Adobe Systems, Mountain View, CA).

### Cell migration and cell invasion assays

For two-dimensional, wound-sealing assays, 2.5 x 10^4^ cells were seeded in each well of 12-well plates and incubated at 37°C. When cells were confluent, the monolayer was wounded in three regions with a sterile pipette tip. Cell debris was washed out with ice-cold PBS, followed by addition of regular culture medium. Phase-contrast images from three zones of each wound were collected immediately after the wounding, and after 8–24 h of incubation at 37°C. Images were acquired with an AxioObserver.D1 microscope equipped with an A-Plan 5x objective (NA 0.12; Ph0), and an AxioCam MRm digital camera, using AxioVision software (Carl Zeiss). The area of wound sealing was quantified using ImageJ software (version 1.47h; [52]). For migration assays in Boyden chambers, we used polycarbonate transwells in 24-well plates, with a pore size of 8.0 μm (Millipore). The top of each transwell was seeded with 8 x 10^3^ cells in serum-free medium, and to the bottom of the chambers it was added medium containing 10% FBS. After 16 h, cells that migrated to the underside of the transwells were fixed with ice-cold methanol, washed twice with ice-cold PBS, and stained with crystal violet dye. For invasion assays in Boyden chambers, we used polyethylene terephthalate transwells in 24-well plates, with a pore size of 8.0 μm, and coated with Matrigel Matrix (Corning). The top of each transwell was seeded with 8 x 10^3^ cells in serum-free medium, and to the bottom of the chambers it was added medium containing 10% FBS. After 16 h, transwell membranes were processed as mentioned before. Images of crystal violet dyed transwell membranes from both migration and invasion assays were acquired with an Axiovert 40 CFL microscope equipped with EC Plan-Neofluar 10x (NA 0.3) and A-Plan 40x (NA 0.65; Ph2 Var2) objectives, and an AxioCam MRc5 digital camera using AxioVision software (Carl Zeiss). For time-lapse, wound-healing assays analysis, cells were seeded in 35-mm glass-bottom culture dishes (MatTek) and incubated at 37°C. When cells were confluent, the monolayer was wounded once with a sterile pipette tip. After washing out cell debris with ice-cold PBS, regular culture medium was added, and the culture dishes were transferred to a microscopy heating stage equipped with temperature, humidity and CO_2_ comptrollers (Carl Zeiss). Images were acquired every 5-min with an AxioObserver.D1 microscope equipped with an A-Plan 5x objective (NA 0.12; Ph0), and an AxioCam MRm digital camera using AxioVision software (Carl Zeiss). The movement of cells from consecutive images was manually tracked using the plug-in Manual Tracking developed for ImageJ software (http://rsb.info.nih.gov/ij/plugins/track/track.html, Fabrice P. Cordelières, Institut Curie, France). Quantitative analysis of cell migration parameters was performed using the program DiPer, as described [54]. To prepare figures, single frames were processed with Adobe Photoshop CS3 (Adobe Systems). QuickTime movies were generated using ImageJ software.

### Cell transfection and time-lapse fluorescence microscopy

Cells were seeded in 35-mm glass-bottom culture dishes (MatTek) and incubated at 37°C. When cells were ~60% confluent, a DNA construct encoding mCherry-tagged vinculin (mCherry-vinculin) [55] was used for transient transfections performed with Lipofectamine 2000 (Life Technologies), according to the manufacturer's instructions. After 16-h, transfected cells were transferred to a microscopy heating stage equipped with temperature, humidity and CO_2_ comptrollers (Live Cell Instrument). Time-lapse, single z-slice images were acquired with a spinning-disk microscope (Leica) equipped with a PlanApo 63x oil immersion objective (NA 1.4; Leica) and an iXon Ultra 888 EMCCD camera (Andor), illuminating with a 20-mW 561-nm laser diode (Andor), and using MetaMorph NX microscopy automation software (Molecular Devices). Kymograph analysis of mCherry-vinculin puncta was performed with ImageJ software using the plug-in MultipleKymograph (http://imagej.net/Multi_Kymograph). Quantification of focal adhesion persistence was performed by manually tracking the length (time) that a mCherry-vinculin (fluorescent) puncta persisted along a kymograph image. For each cell analyzed, five regions of interest were selected for kymograph generation, and for each kymograph the length of four puncta were measured. Time of focal adhesion persistence was normalized to that of control (shLuc) cells. QuickTime movies were generated using ImageJ software.

### Disassembly and reassembly of focal adhesions

We performed a similar focal adhesion disassembly-reassembly assay to one described before [56]. Briefly, cells seeded in glass coverslips were either left untreated or treated with 10 μM nocodazole for 4 h at 37°C. Some cells were also subjected to washout of nocodazole for different periods of time, from 20 min to 2 h, using regular culture medium. Cells were fixed in 4% paraformaldehyde, and processed for fluorescence microscopy as described above, using antibody to vinculin to detect focal adhesions, and TRITC-phalloidin to decorate actin filaments. We performed the quantification of the fluorescence signal associated to focal adhesions by using a method of fluorescence quantification that we have described previously [51]. Briefly, we used an AxioObserver.D1 microscope (Carl Zeiss), equipped as indicated before, to acquire 12-bit images under identical settings, avoiding signal saturation, and corrected for background, crosstalk and noise signals on each set of images. The resulting processed images were transformed to binary images, and we obtained the area occupied by focal adhesions from the total integrated pixel intensity. Each value of total focal adhesions area was normalized to each value of the corresponding cell area. All image processing and analysis was performed using ImageJ software (version 1.47h; [52]).

### Lentiviral transduction

The full-length, coding sequence of human GOLPH3 (GenBank/EMBL/DDBJ accession number NM_022130) was obtained by PCR amplification, using as template a construct encoding GFP-tagged, full-length GOLPH3 [49], and cloned in frame into the *Not*I and *Bam*HI restriction sites of the lentiviral expression vector pLVX-IRES-Puro (Clontech). To generate GOLPH3 lentiviral particles (LV-GOLPH3), we used a method that we have described elsewhere [57]. For both fluorescence microscopy analysis of focal adhesions and wound-sealing migration assays, cells were transduced with LV-GOLPH3 and led to grow for up to 5 days. The levels of GOLPH3 expression after transduction were checked everyday by immunoblot and immunofluorescence, using these methods as indicated before. The maximal effects of GOLPH3 lentiviral expression were observed between 4–5 days of transduction. For experiments undergoing FAK inhibition, cells were transduced, allowed to grow for 3 days, and 2.5 x 10^4^ cells were seeded in each well of 12-well plates. The next day, when cells were confluent, the monolayer was wounded in three regions with a sterile pipette tip. Cell debris was washed out with ice-cold PBS, followed by addition of regular culture medium supplemented with 1 μM of the FAK inhibitor Compound PF-562271. Phase-contrast images from three zones of each wound were collected immediately after the wounding, and after 8–24 h of incubation, as indicated above.

### Statistical analysis

**Fig 1:**
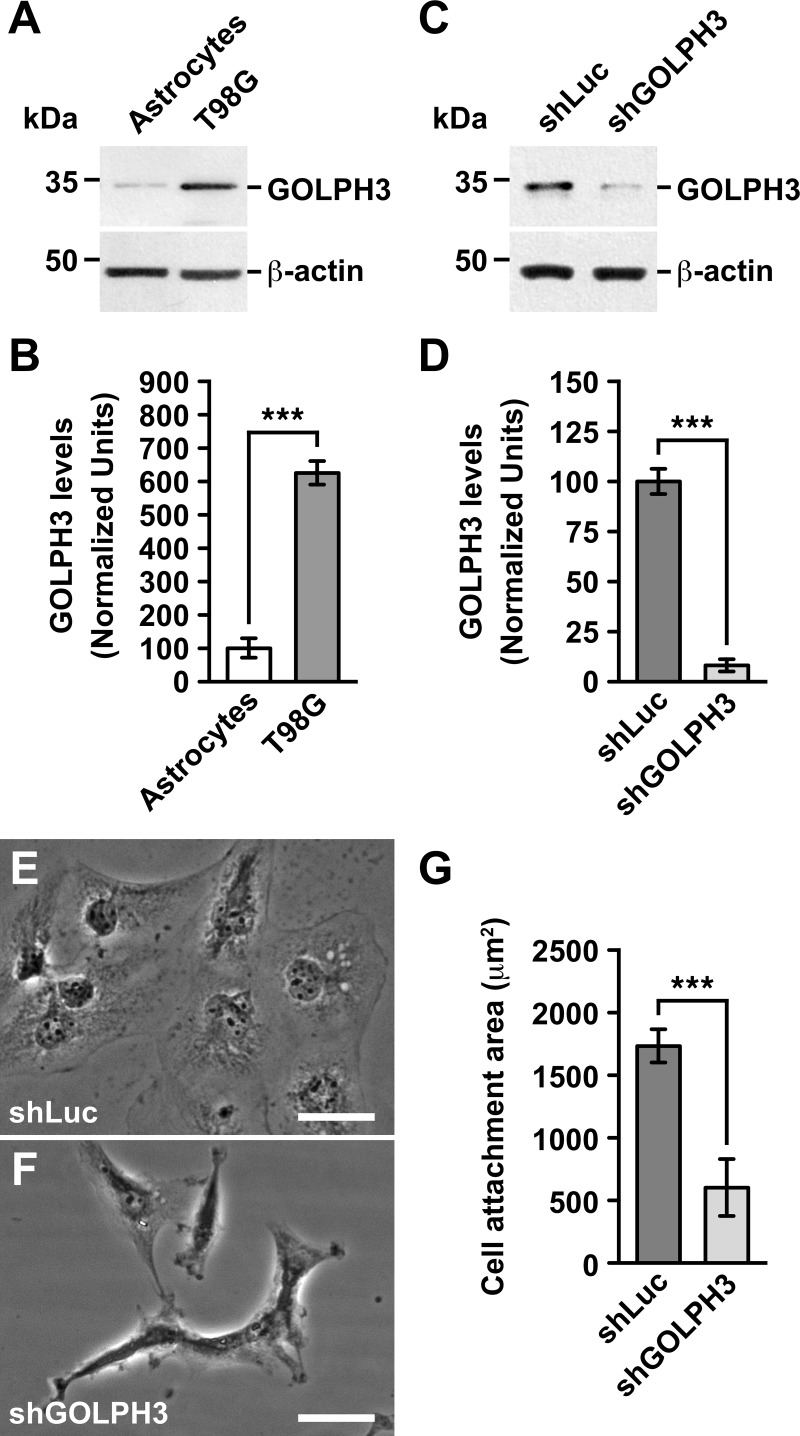
The knocking down of GOLPH3 reduces the cell attachment area of T98G cells. (A-D) Detergent-soluble extracts were prepared from the indicated cells (A and C), and proteins were analyzed by SDS-PAGE followed by immunoblotting using antibodies to the proteins indicated on the right. The immunoblot signal of anti-β-actin was used as loading control. The position of molecular mass markers is indicated on the left. In *B* and *D* is shown a densitometry quantification of the immunoblot signal of the levels of GOLPH3 as shown in *A* and *C*, respectively. Bar represents the mean ± standard deviation of replicates (n = 5). *** *P* < 0.001. (E and F) Phase-contrast images of the indicated cells grown in regular conditions. Bar, 20 μm. (G) Quantification of the area attached to the substratum of the indicated cells as shown in E and F. Bar represents the mean ± standard deviation (n = 15). *** *P* < 0.001.

**Fig 2:**
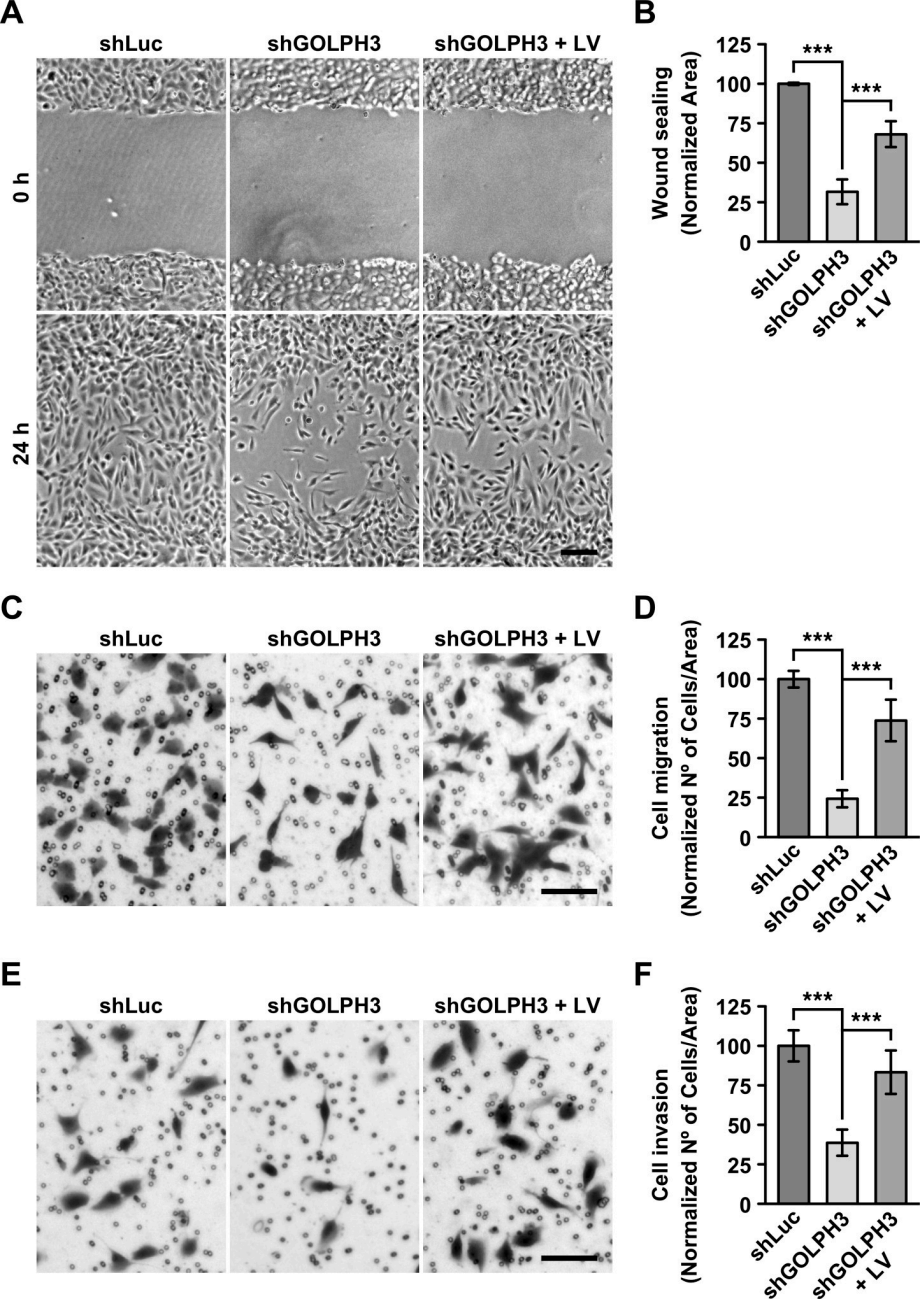
The knocking down of GOLPH3 affects the migration and invasion of T98G cells. (A-F) The indicated cells were left untreated (*shLu*c and *shGOLPH3*) or subjected to lentiviral transduction for 5 days to express RNAi-resistant GOLPH3 (*shGOLPH3 + LV*). (A) Confluent cultures of the indicated cells grown in 12-well plates were wounded with a sterile tip. Phase-contrast images of the same regions of the wounds were taken immediately after the wounding (*0 h*), and after 24-h (*24 h*). Bar, 100 μm. (B) Quantification of cell migration from images as shown in *A*, estimated as the area re-occupied by cells after 24-h. (C) Images of the indicated cells seeded on top of transwells of Boyden chambers, grown for 16-h, and stained with crystal violet dye. Bar, 100 μm. (D) Quantification of the cell migration from images as shown in *C* was estimated as the number of cells detected in the underside of the transwells after 16-h. (E) Images of the indicated cells seeded on top of Matrigel Matrix-coated transwells of Boyden chambers, grown for 16-h, and stained with crystal violet dye. Bar, 100 μm. (F) Quantification performed as described in *D* of cell invasion from images as shown in *E*. Bar represents the mean ± standard deviation (n = 3). *** *P* < 0.001.

**Fig 3:**
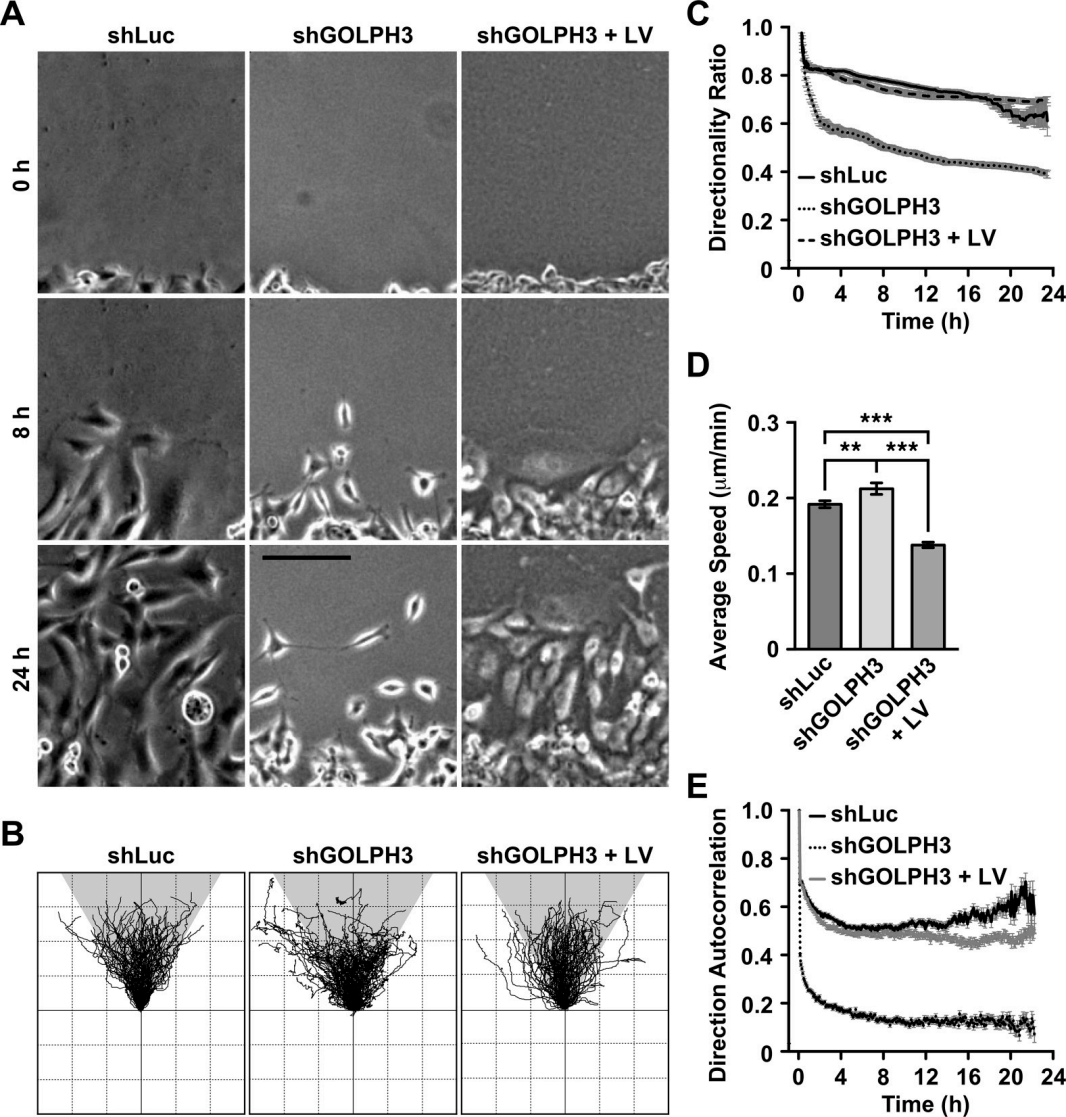
The knocking down of GOLPH3 affects directional persistence of T98G cells. (A-E) The indicated cells were left untreated (*shLu*c and *shGOLPH3*) or subjected to lentiviral transduction for 5 days to express RNAi-resistant GOLPH3 (*shGOLPH3 + LV*). (A) Confluent cultures of the indicated cells grown in 35-mm glass-bottom culture dishes were wounded with a sterile tip. The dishes were transferred to a microscopy heating stage equipped with temperature, humidity and CO_2_ comptrollers, and phase-contrast images were acquired immediately, and every 5-min up to 24 h. Representative images acquired at the indicated times are shown. Bar, 50 μm. (B-E) The movement of cells from consecutive images of the time-lapse microscopy shown in *A* was manually tracked, and each trajectory (n = 100 for each cell line) was plotted with the same origin in a Cartesian coordinate system (B). The directionality ratio (C), average speed (D), and direction autocorrelation (E) parameters were obtained using DiPer software. Bar represents the mean ± standard deviation (n = 100). ** *P* < 0.01; *** *P* < 0.001.

**Fig 4:**
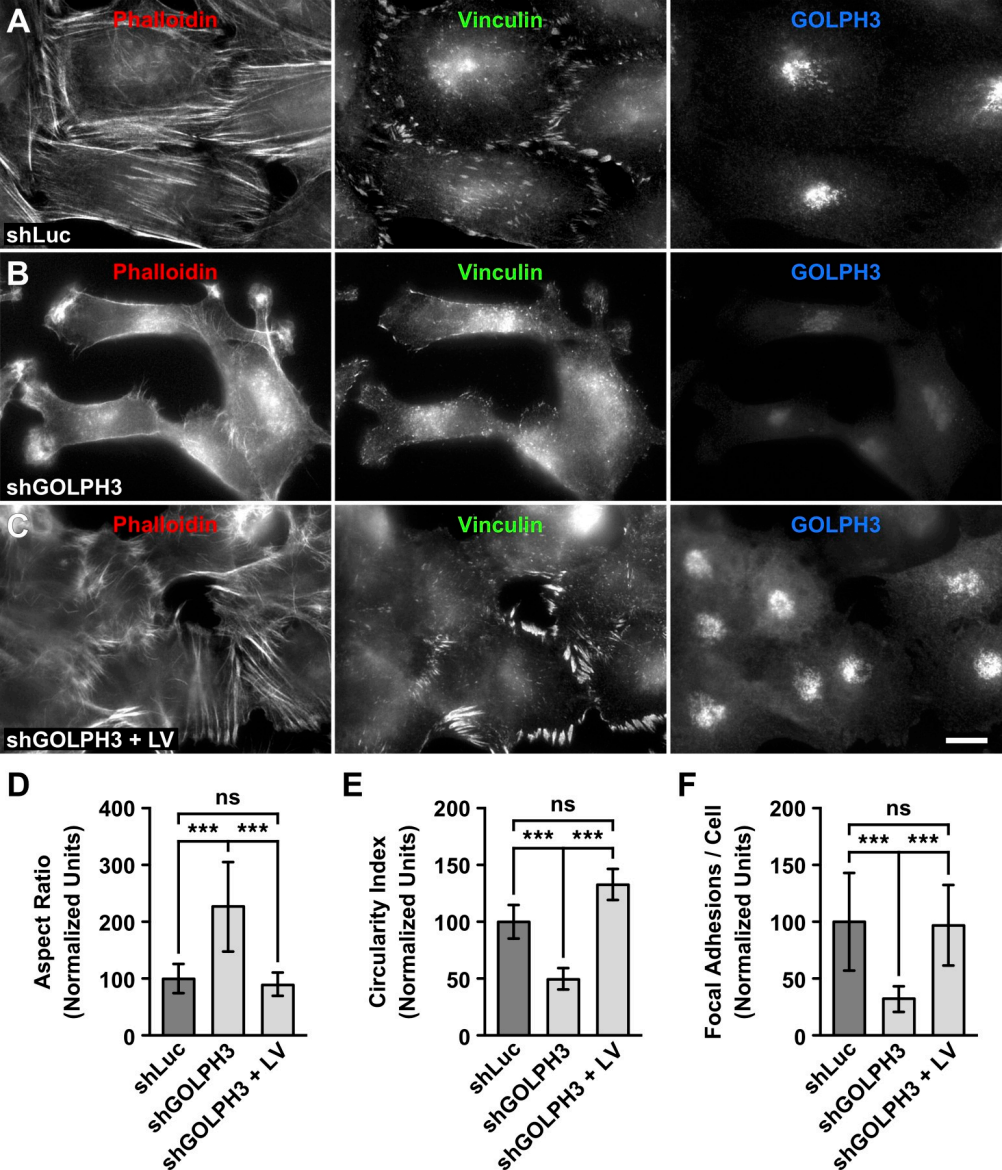
The knocking down of GOLPH3 affects focal adhesions of T98G cells. (A-C) The indicated cells grown in glass coverslips were left untreated (A and B) or subjected to lentiviral transduction for 5 days to express RNAi-resistant GOLPH3 (C). Cells were fixed, permeabilized, and triple-labeled with mouse monoclonal antibody to vinculin, rabbit polyclonal antibody to GOLPH3, and TRITC-phalloidin. Secondary antibodies were Alexa-488-conjugated donkey anti-mouse IgG and Alexa-647-conjugated donkey anti-rabbit IgG. Stained cells were examined by fluorescence microscopy. Bar, 10 μm. (D-F) Quantification of the cell shape parameters aspect ratio (D; n = 35) and circularity index (E; n = 35), and of focal adhesions (F; n = 15), performed as indicated in *Materials and Methods*, from images as shown in A-C. Bar represents the mean ± standard deviation. *** *P* < 0.001; *ns*, not statistically significant.

**Fig 5:**
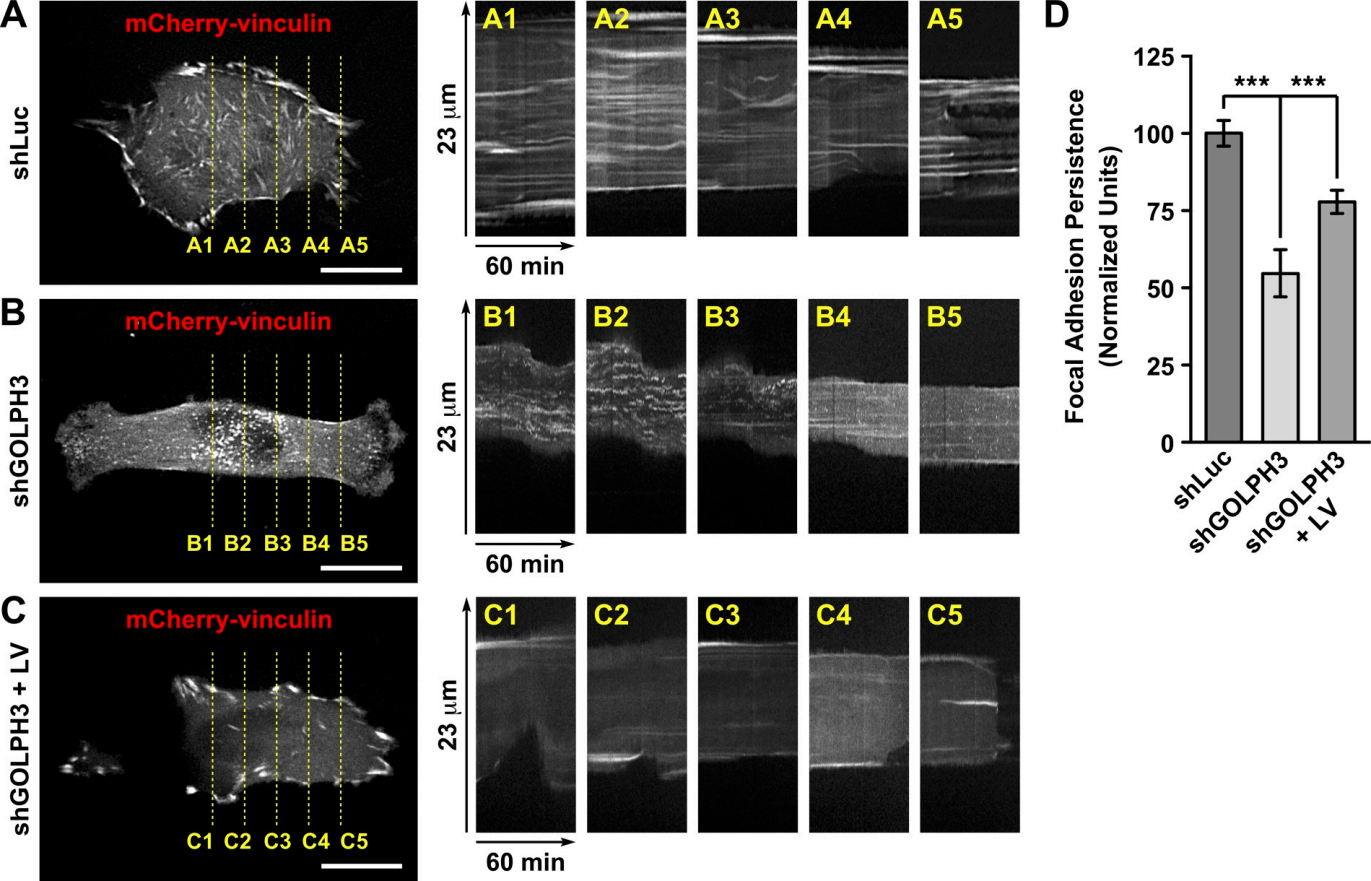
The knocking down of GOLPH3 affects focal adhesion dynamics of T98G cells. (A-C) The indicated cells were left untreated (*shLu*c and *shGOLPH3*) or subjected to lentiviral transduction for 5 days to express RNAi-resistant GOLPH3 (*shGOLPH3 + LV*). After 3 days, cells were seeded in 35-mm glass-bottom culture dishes, and after 24-h cells were transfected with a DNA construct encoding mCherry-tagged vinculin (*mCherry-vinculin*). After 16-h, the dishes were transferred to a microscopy heating stage equipped with temperature, humidity and CO_2_ comptrollers, and fluorescent images were acquired every 30-sec. The left panels show the last image of each time-lapse acquisition. The right panels (A1-A5, B1-B5 and C1-C5) show kymographs depicting the indicated period of time of image acquisition, and length of the image sections highlighted in the respective left panels. (C) Quantification of the time of persistence of fluorescent puncta during the time of image acquisition from kymographs as shown in *A*, *B* and *C*. Bar represents the mean ± standard deviation (quantification of 20 fluorescent puncta per cell; n = 10 cells). *** *P* < 0.001.

**Fig 6:**
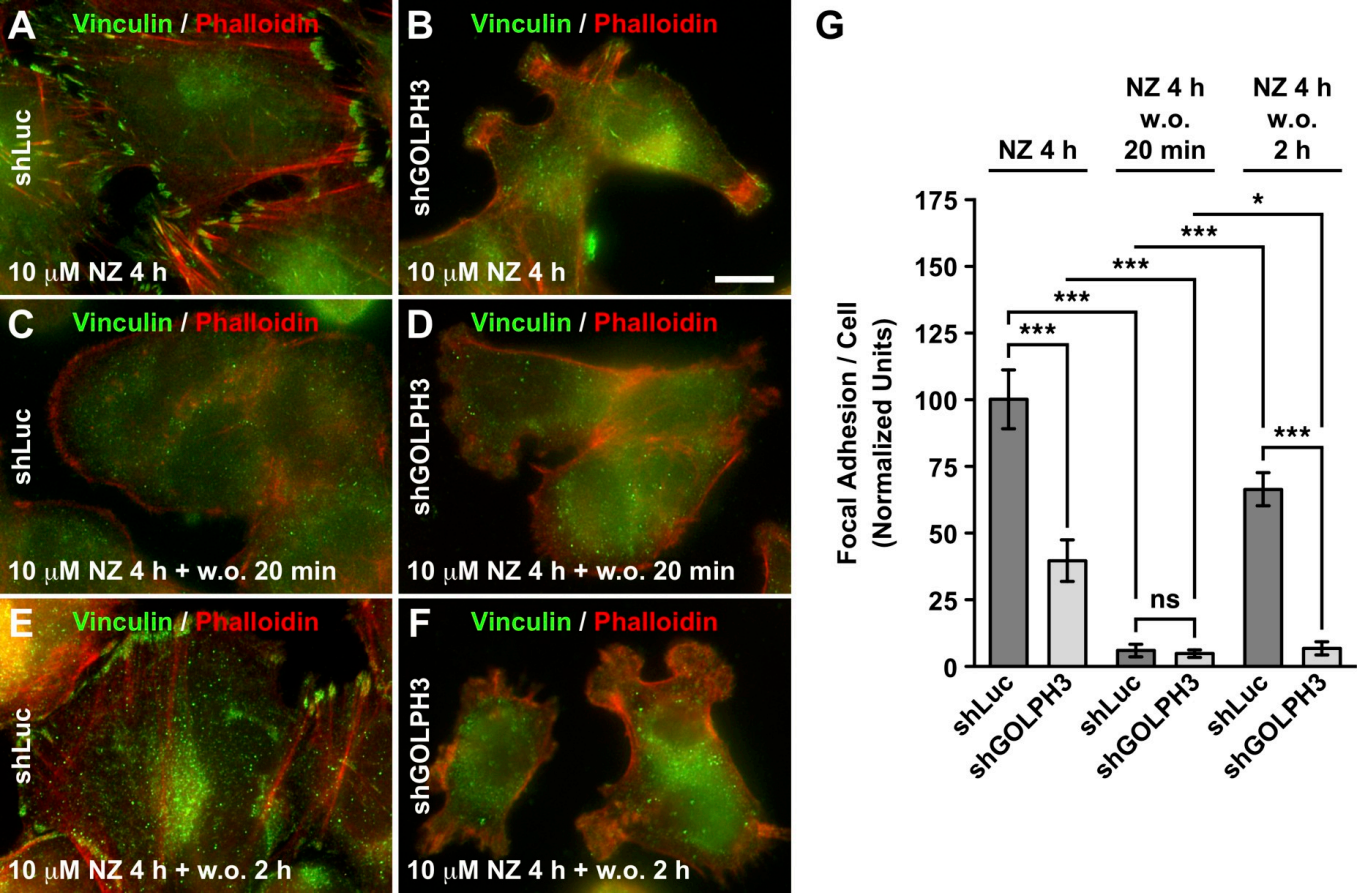
The knocking down of GOLPH3 affects the reassembly of focal adhesion in T98G cells. (A-F) The indicated cells grown in glass coverslips were treated with 10 μM nocodazole for 4 h at 37°C (A and B), or subsequently subjected to nocodazole washout for either 20 min (C and D) or 2-h (E and F) at 37°C. Cells were fixed, permeabilized, and double-labeled with mouse monoclonal antibody to vinculin, and TRITC-phalloidin, followed by Alexa-488-conjugated donkey anti-mouse IgG. Stained cells were examined by fluorescence microscopy. Bar, 10 μm. (G) Quantification of focal adhesions of the indicated cells subjected to the indicated treatments from images as shown in *A*-*F*, performed as indicated in *Materials and Methods*. Bar represents the mean ± standard deviation (n = 30 cells). * *P* < 0.05; *** *P* < 0.001; *ns*, not statistically significant.

**Fig 7:**
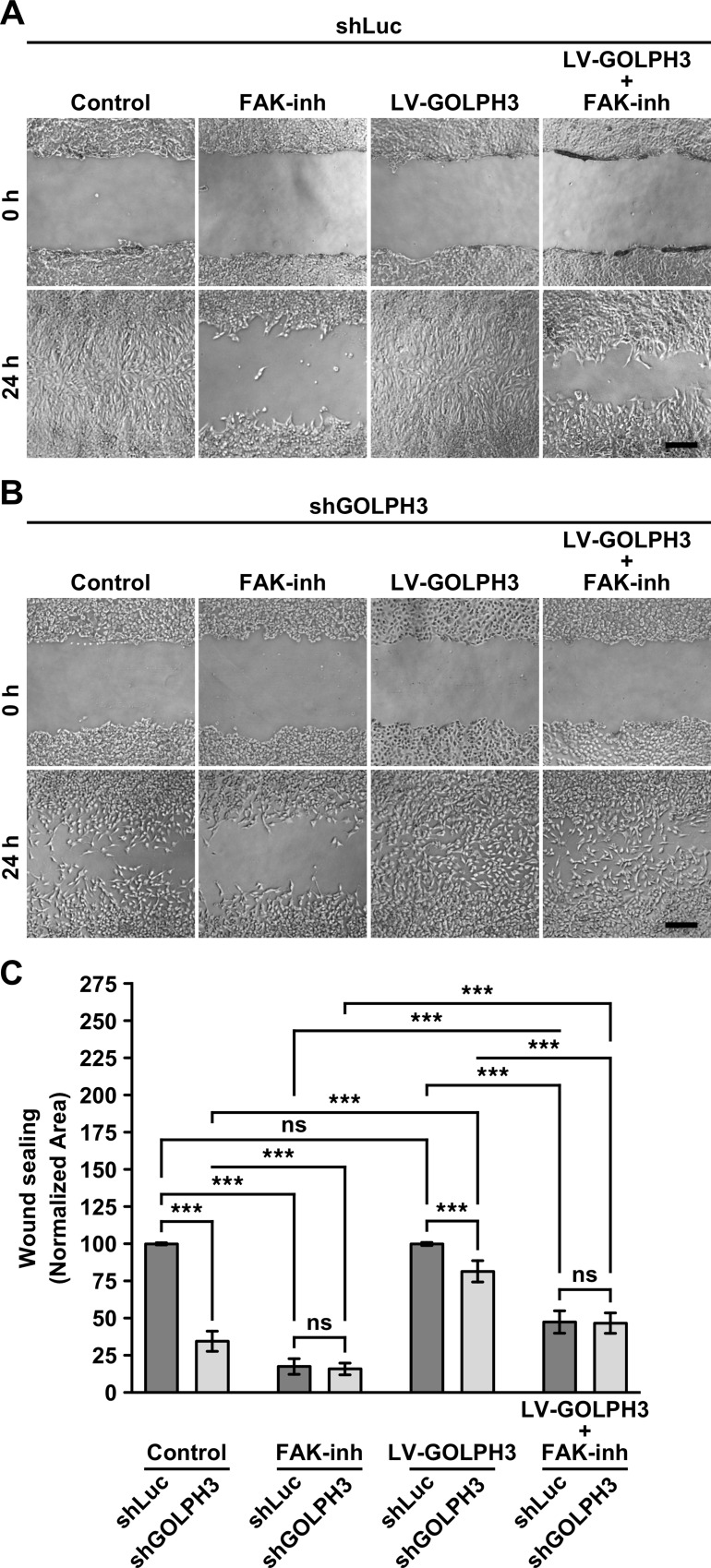
The knocking down of GOLPH3 affects the migration of T98G cells in a FAK-dependent manner. (A and B) The indicated cells grown in 12-well plates were left untreated, or subjected to lentiviral transduction for 4 days to express GOLPH3 (LV-GOLPH3). The monolayers of confluent cells were wounded in three regions with a sterile pipette tip, and further left untreated (*Control* and *LV-GOLPH3*), or treated with 1 μM of the FAK inhibitor Compound PF-562271 for 24-h (*FAK-inh* and *LV-GOLPH3 + FAK-inh*). Phase-contrast images of the same regions of the wounds were taken immediately after the wounding (*0 h*), and after 24-h (*24 h*). Bar, 100 μm. (C) Quantification of cell migration from images as shown in *A* and *B*, estimated as the area re-occupied by cells after 24-h. Bar represents the mean ± standard deviation (n = 3). *** *P* < 0.001; *ns*, not statistically significant.

**Fig 8:**
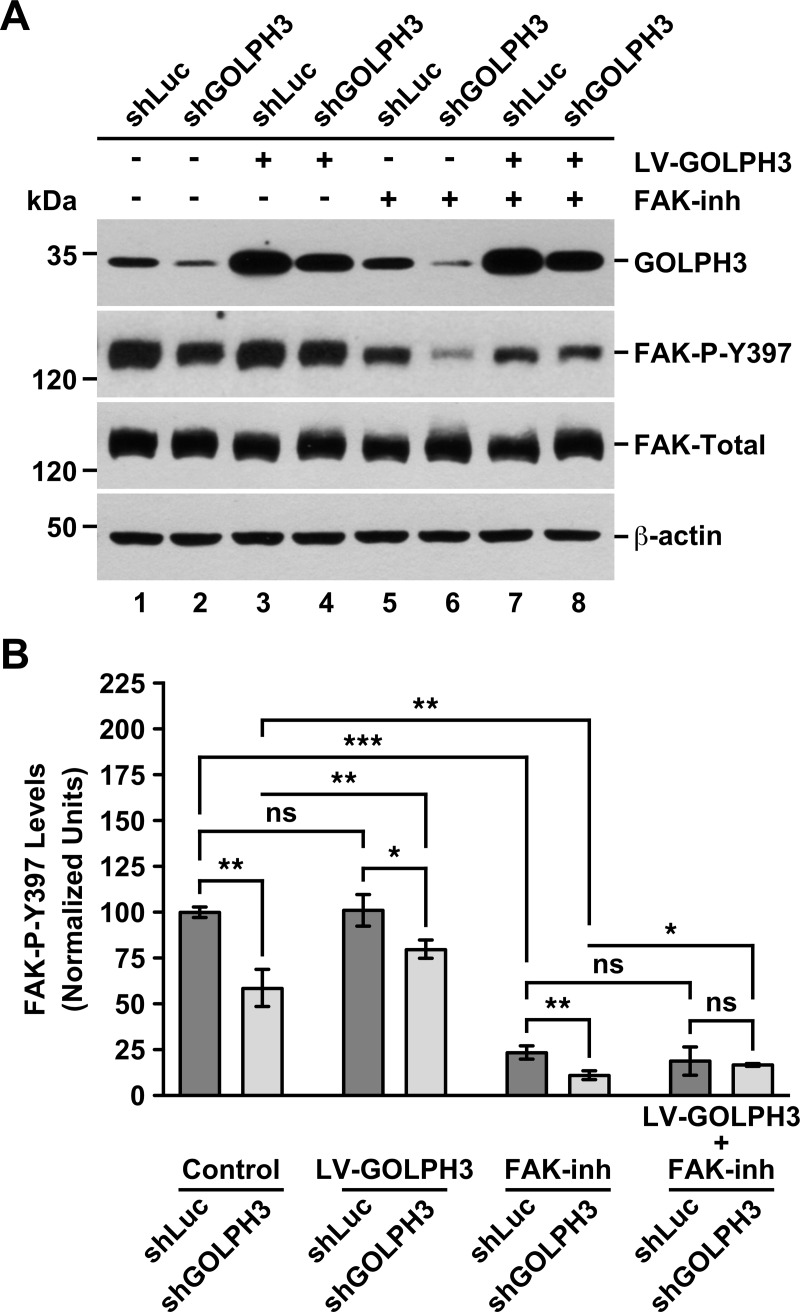
The knocking down of GOLPH3 in T98G cells affects the phosphorylation of FAK on Tyr-397. (A) The indicated cells were left untreated (lanes 1 and 2), or either subjected to lentiviral transduction for 5 days to express GOLPH3 (*LV-GOLPH3*; lanes 3 and 4), or treated with 1 μM of the FAK inhibitor Compound PF-562271 for 24-h (*FAK-inh*; lanes 5 and 6), or subjected to lentiviral transduction for 4 days to express GOLPH3 followed by further treatment with 1 μM of the FAK inhibitor for 24-h (lanes 7 and 8). Detergent-soluble extracts were prepared, and proteins were analyzed by SDS-PAGE followed by immunoblotting using antibodies to the proteins indicated on the right. The immunoblot signal of anti-β-actin was used as loading control. The position of molecular mass markers is indicated on the left. *FAK-P-Y397*: FAK phosphorylated on Tyr-397. The panels show representative images. (B) Densitometry quantification of the immunoblot signal of the levels of GOLPH3 as shown in *A*. Bar represents the mean ± standard deviation (n = 3). *** *P* < 0.001; *ns*, not statistically significant.

Statistical analysis was performed using Microsoft Excel for Mac 2011 (Microsoft Corporation). When appropriate, results were represented in graphs depicting the mean ± standard deviation. Statistical significance was determined by two-tailed, paired *t*-test. *P*-values > 0.05 or *≤* 0.05 were regarded as not statistically significant or statistically significant, respectively. In Figs 1–8, and S1–S3 Figs, *P*-values between 0.01 and 0.05 are indicated with one asterisk, *P*-values between 0.001 and 0.01 are indicated with two asterisks, and *P*-values less than 0.001 are indicated with three asterisks.

## Results

### The knocking down of GOLPH3 disrupts cell migration and cell invasion of T98G cells

Several reports have shown that GOLPH3 is overexpressed in different human tumor tissues, including gliomas [19, 29, 58, 59], as well as in human glioma cell lines such as U87, U118 and U251 cells [29, 59]. However, although T98G cells have been broadly used as a model of glioblastoma multiforme [60–62], and that they seem to have acquired a unique transformation mechanism [63], it is unknown whether GOLPH3 plays a role in the tumorigenic features of these cells. Thus, we first compared by immunoblot analysis the level of expression of GOLPH3 in T98G cells to that of human astrocytes in primary culture. We found significant, ~ 6-fold higher levels of GOLPH3 in T98G cells (Fig 1A and Fig 1B), indicating that this cell line is suitable to study the functional effects of reducing the expression of GOLPH3. To this aim, we generated T98G cells stably expressing an shRNA to luciferase (shLuc cells; used as negative control), or either of two shRNA to GOLPH3 (shGOLPH3#1 and shGOLPH3#2). Because the results obtained were very similar irrespective of the shRNA used for the knocking down of GOLPH3 expression, for the sake of simplicity below are shown only the results of cells expressing shGOLPH3#1, which are regarded as shGOLPH3 cells. As expected, the levels of GOLPH3 in wild type and shLuc cells were indistinguishable, as shown by immunoblot analysis (S1 Fig). Instead, the levels of GOLPH3 in shGOLPH3 cells were significantly reduced, as shown by immunoblot (Fig 1C and Fig 1D) and immunofluorescence (S2 Fig) analyses. Moreover, the levels of GOLPH3 in shGOLPH3 cells were similar to those found in astrocytes (although significantly lower; Fig 1A, Fig 1C, and S3 Fig), rendering shGOLPH3 cells appropriate to study the functional effects of knocking down GOLPH3 in T98G cells.

Because GOLPH3 seems to play tumorigenic roles in part through membrane trafficking processes [45], our primary focus of study was that of proteins related to oncogenic transformation, such as those involved in signal transduction pathways. However, we unexpectedly observed a dramatic change in the morphology of shGOLPH3 cells. This observation led us instead to analyze first in more detail this phenotype to determine whether it correlated with changes in tumorigenic features. We found that while wild type and shLuc cells showed an amoeboid shape (Fig 1E), shGOLPH3 cells resembled a mesenchymal phenotype with multiple lamellae (Fig 1F), morphology that resulted in a significant reduction of cell attachment area (Fig 1G). This change in morphology of shGOLPH3 cells suggested that the knocking down of GOLPH3 expression would affect the motility of T98G cells. To assess this possibility, we performed a wound-sealing assay. We found that after 24-h the area of the wounds covered by shGOLPH3 cells was significantly reduced to 31.6 ± 7.9% the area covered by control cells (Fig 2A and Fig 2B), suggesting that the knocking down of GOLPH3 expression greatly affects cell migration. Expression of RNAi-resistant GOLPH3 by lentivirus-transduction of shGOLPH3 cells showed a significant increase in the wound area covered by these cells (Fig 2A and Fig 2B). Although the wound area covered by GOLPH3-expressing shGOLPH3 cells reached 68.1 ± 8.2% that of control cells, these results indicate that the levels of GOLPH3 modulate the level of cell migration in T98G cells. To get additional evidence of the effect that the knocking down of GOLPH3 expression has on cell motility, we performed a migration assay in Boyden chambers. We found that after 16-h the amount of shGOLPH3 cells observed in the bottom of transwell membranes of Boyden chambers was significantly reduced to 24.3 ± 5.4% the level reached by control cells (Fig 2C and Fig 2D). This result correlates well with the result of the wound-sealing assay, corroborating that the knocking down of GOLPH3 affects the migration of T98G cells. Expression of RNAi-resistant GOLPH3 resulted in a significant increase in the amount of shGOLPH3 cells in the bottom of transwell membranes (Fig 2C and Fig 2D). Likewise, although the amount of migrating, GOLPH3-expressing shGOLPH3 cells reached 73.9 ± 13.2% that of control cells, these results corroborate that the migration of T98G cells is modulated by the levels of GOLPH3. Because cell migration and cell invasion are related tumor progression features, we next performed an invasion assay in Boyden chambers with transwells coated with Matrigel. We found that after 24-h the number of shGOLPH3 cells detected in the bottom of transwell membranes were significantly reduced, to 38.6 ± 8.3% compared to control cells (Fig 2E and Fig 2F). Expression of RNAi-resistant GOLPH3 increased the invasion of shGOLPH3 cells to reach 83.3 ± 13.8% the levels found in control cells, indicating that the levels of GOLPH3 also modulate cell invasion in T98G cells. Together, these results indicate that the knocking down of GOLPH3 negatively affects both cell migration and cell invasion of T98G cells.

### The knocking down of GOLPH3 disrupts directional persistence during migration of T98G cells

The peculiar morphology of shGOLPH3 cells suggested that this feature could be related to the observed reduced efficiency of cell migration and cell invasion. To analyze this possibility, we performed a wound-sealing assay tracking cell migration by video-microscopy during 24-h. From the visual inspection of cell migration it was readily apparent that, in contrast to control cells, shGOLPH3 cells migrated with less directional persistence (S1 Video). Moreover, we observed that during migration, the majority of shGOLPH3 cells projected more than one migration protrusion, which were eventually projected at slightly different times, toward different directions, and often reaching different lengths (Fig 3A, S1 Video and S4 Fig). The migration of shGOLPH3 cells continued with cell retractions from the opposite side of each protrusion, some times unsynchronized, and followed by new rounds of protrusions projection, eventually toward different directions than the preceding projections (S1 Video and S4 Fig). This behavior very often resulted in dramatic changes in the direction of migration (S1 Video and S5 Fig). On the other hand, some shGOLPH3 cells reached a point in which the rounds of protrusions' projection seemed more synchronized to the corresponding retractions. This resulted in stationary behavior, with apparent little persistence of migration (S1 Video and S6 Fig). The difference in directional persistence was more apparent when we compared individual cell migration tracks, showing that those from shGOLPH3 cells were more convoluted than those of control cells (Fig 3B). The general behavior of shGOLPH3 cells during migration explains the peculiar morphology observed in fixed cells (Fig 1F), and suggests that it could be related to impaired cell polarity. In addition, this behavior strongly suggests that the directional persistence of cell migration was disrupted. A further quantitative analysis supported this last notion. We found a significant reduction of migration persistence (directionality ratio) throughout the time of migration of shGOLPH3 cells (Fig 3C). Reduction in migration persistence often is explained by reduction in migration speed. However, although moderate, we found a significant increase in the speed of migration of shGOLPH3 cells (Fig 3D). Nevertheless, a direction autocorrelation analysis showed a significant reduction in the directionality of migration of shGOLPH3 cells (Fig 3E). Expression of RNAi-resistant GOLPH3 by lentivirus-transduction of shGOLPH3 cells resulted in a striking change in motility behavior, similar to that of control cells, with significant increase in both migration persistence (Fig 3C) and directionality of migration (Fig 3E). Moreover, we also found a significant decrease in the speed of migration of GOLPH3-expressing shGOLPH3 cells that was even significantly lower compared to that of control cells (Fig 3D). Thus, together, these results indicate that the knocking down of GOLPH3 expression resulted in a reduction of directional persistence during migration of T98G cells, and that overall the levels of GOLPH3 modulate the motility of these cells.

### The knocking down of GOLPH3 disrupts focal adhesions in T98G cells

The change in morphology of shGOLPH3 cells suggested a rearrangement of their actin cytoskeleton. On the other hand, the cell migration behavior of shGOLPH3 cells suggested a distinct adhesion to the substratum. To test these possibilities we performed fluorescence microscopy analysis. To detect elements of the actin cytoskeleton we used TRITC-conjugated phalloidin, and to detect focal adhesions we performed immunofluorescence with antibodies to vinculin. In control cells, we found a robust phalloidin decoration of stress fibers (Fig 4A). In contrast, shGOLPH3 cells showed little decoration of stress fibers. Instead, we found robust phalloidin decoration of structures reminiscent of membrane ruffles, and in all the protrusions of each cell (Fig 4B). This observation suggested that the rearrangement of the actin cytoskeleton led the shape change of shGOLPH3 cells, as indicated by a significant increase in their aspect ratio (Fig 4D), and a significant decrease in their circularity index (Fig 4E). On the other hand, the immunofluorescence analysis showed that while vinculin was robustly detected at the tip of stress fibers in control cells (Fig 4A), in shGOLPH3 cells it was detected in significantly fewer and smaller puncta (Fig 4B and Fig 4F), suggesting that the knocking down of GOLPH3 expression disrupted focal adhesions of T98G cells. To test this possibility, we expressed RNAi-resistant GOLPH3 by lentivirus-transduction of shGOLPH3 cells. Strikingly, we first observed that the shape of shGOLPH3 cells changed to a type that was indistinguishable to that of control cells (Fig 4C and Fig 4E). This apparent reversion of shape correlated with the presence of robust stress fibers, and the recovery of focal adhesions (Fig 4C and Fig 4F).

### The knocking down of GOLPH3 disrupts the dynamics of focal adhesions in T98G cells

The behavior of shGOLPH3 cells during migration, as well as the immunofluorescence detection of vinculin, suggested a distinct dynamics of focal adhesions in these cells. To test this hypothesis, we first analyzed the expression of mCherry-vinculin by time-lapse, fluorescence microscopy in living cells. We observed that mCherry-vinculin was incorporated in puncta that resembled very well those observed containing endogenous vinculin, in either control cells (Fig 4A and Fig 5A) or shGOLPH3 cells (Fig 4B and Fig 5B). A kymograph analysis of control cells showed that mCherry-vinculin remained mostly stably associated to the puncta during the entire period of image acquisition (Fig 5A and S2 Video), indicating little disassembly of focal adhesions. In contrast, the kymograph analysis of shGOLPH3 cells showed a more dynamic behavior of mCherry-vinculin (Fig 5B and S2 Video). Quantification of this dynamic behavior showed a significant decrease in the time that mCherry-vinculin remained associated to puncta of shGOLPH3 cells, to 54.7 ± 7.6% compared to the time of stability found in control cells (Fig 5D). Expression of RNAi-resistant GOLPH3 by lentivirus-transduction of shGOLPH3 cells showed that mCherry-vinculin was incorporated in focal adhesion puncta that were very similar to those of control cells (Fig 5C). Importantly, these puncta remained largely stable, increasing to 77.9 ± 3.8% the time of stability found in control cells (Fig 5C and Fig 5D). These results indicate that the levels of GOLPH3 modulate the dynamics of focal adhesion in T98G cells. Also, these results suggest that focal adhesions in shGOLPH3 cells are short-lived, or that their assembly is less efficient. To distinguish between these possibilities, we performed a microtubule-dependent, focal adhesion disassembly-reassembly fluorescence microscopy assay [56]. In this assay, serum-starved cells are treated for 4 h with 10 μM nocodazole, which results in nocodazole inhibition of microtubule polymerization [64], and subsequent stabilization of focal adhesions [65]. The assay follows with nocodazole washout, which leads to the disassembly of focal adhesions during partial microtubule regrowth, ending in reassembly of focal adhesions after 2 h of nocodazole washout [56]. As expected, in control cells treated with nocodazole we observed lack of microtubules, as well as robust focal adhesions, as indicated by immunofluorescence detection of α-tubulin (S7 Fig), and of vinculin in the tips of phalloidin-decorated stress fibers (Fig 6A). In contrast, shGOLPH3 cells showed fewer and smaller puncta detected with antibodies to vinculin (Fig 6B), indicating a reduction in the number of focal adhesions. Similar to the condition of growing cells in serum-supplemented medium (Fig 4), we found that the reduction of focal adhesions in shGOLPH3 cells at the beginning of the disassembly-reassembly assay was significant (Fig 6G). On the other hand, after 20 min of nocodazole washout, we observed a dramatic and significant reduction of puncta detected with antibodies to vinculin, in both control and shGOLPH3 cells (Fig 6C, Fig 6D and Fig 6G), which is consistent with a reduction of focal adhesions during microtubule regrowth [56]. After 2 h of nocodazole washout, and compared to the levels reached after 20 min of washout, we observed in control cells a significant, ~10.5-fold increase of puncta detected with antibodies to vinculin (Fig 6E and Fig 6G), consistent with the expected reassembly of focal adhesions [56]. However, in shGOLPH3 cells, we found an increase of puncta to a much lesser extent (Fig 6F and Fig 6G). In fact, although it was significant, compared to the levels reached after 20 min of nocodazole washout, it represented ~2.5-fold increase (Fig 6G), indicating less efficient focal adhesions reassembly. Thus, together with the live-cell imaging analysis, these results strongly indicate that the knocking down of GOLPH3 expression disrupts the dynamics of focal adhesions in T98G cells.

### GOLPH3 promotes the migration of T98G cells via focal adhesion kinase activity

The dynamics of focal adhesions formation and disassembly in migrating cells is associated to the activity of FAK [66–68]. Thus, we hypothesized that the less efficient migration of shGOLPH3 cells could be related to FAK activity. To test this hypothesis, we evaluated whether or not the less efficient migration of shGOLPH3 cells could be reverted by lentiviral expression of RNAi-resistant GOLPH3 in a FAK activity-dependent manner. To this, we performed a wound-sealing assay. As shown before (Fig 2A), we found that after 24 h the migration of shGOLPH3 cells were significantly less efficient than of control cells (Fig 7A and Fig 7C). As expected, we found that incubation with the FAK inhibitor Compound PF-562271 resulted in a significantly reduction of the respective migration of control and shGOLPH3 cells to 17.0 ± 5.3% and 15.8 ± 3.9%, respectively, of the level found in control cells subjected to mock treatment (Fig 7A and Fig 7C). Importantly, although the expression of RNAi-resistant GOLPH3 in control cells showed no additional migration efficiency, i.e., after 24 h migrating cells covered the entire area of the wound (Fig 7A and Fig 7C), in shGOLPH3 cells resulted in a significant increase of migration efficiency, from 34.8 ± 6.6% in control conditions to 81.2 ± 7.0% (Fig 7B and Fig 7C), indicating that GOLPH3 promotes the migration of T98G cells. Moreover, incubation with the FAK inhibitor showed a significant reduction of migration efficiency of control cells expressing RNAi-resistant GOLPH3, to 47.3 ± 7.7% (Fig 7A and Fig 7C), which was although significantly higher compared to control cells incubated only with the FAK inhibitor (Fig 7A and Fig 7C), suggesting that FAK activity is necessary for the effect of GOLPH3 in the migration of T98G cells. Likewise, incubation with the FAK inhibitor resulted in significant reduction of the effect of the expression of RNAi-resistant GOLPH3 in shGOLPH3 cells, i.e., resulted in a change of migration efficiency from 81.2 ± 7.0% to 46.7 ± 6.8%, which was also significantly higher compared to shGOLPH3 cells incubated only with the FAK inhibitor (Fig 7B and Fig 7C), indicating that GOLPH3 promotes migration of T98G cells via FAK activity.

### The knocking down of GOLPH3 in T98G cells affects phosphorylation of FAK on Tyr-397

The dynamics and disassembly of focal adhesions is regulated by activation of FAK through autophosphorylation at Tyr-397 (Y397; [69]). Thus, we analyzed by immunoblot the levels of phosphorylated FAK at Y397 (Phospho-Y397). Unexpectedly, under mock treatment conditions, we found a significant reduction in the basal levels of Phospho-Y397 in shGOLPH3 cells to 58.6 ± 10.1% the levels found in control cells (Fig 8A, lanes 1 and 2, and Fig 8B, *Control*). This observation suggests that the less efficient migration of shGOLPH3 cells was in fact the result of less activated FAK, and hence that GOLPH3 promotes activation of FAK. Consistent with this hypothesis, compared to mock treatment, we found a significant increase of 36.0 ± 8.5% in the levels of Phospho-Y397 in shGOLPH3 cells subjected to lentiviral expression of RNAi-resistant GOLPH3 (or from 58.6 ± 10.1% to 79.7 ± 5.0% when the levels are compared to the basal levels found in control cells; Fig 8A, lanes 1, 2 and 4, and Fig 8B). Nevertheless, we found that in this condition the levels of Phospho-Y397 in shGOLPH3 cells were still significantly lower than in control cells (Fig 8A, lanes 3 and 4, and Fig 8B, *LV-GOLPH3*), suggesting that complete recovery to control cell-levels of FAK activation requires one or more additional limiting elements. Interestingly, compared to mock treatment, lentiviral expression of RNAi-resistant GOLPH3 in control cells resulted in non-significant differences in the levels of Phospho-Y397 (Fig 8A, lanes 1 and 3, and Fig 8B), suggesting that the effect in the activation of FAK in T98G cells is maximal at the endogenous levels of GOLPH3.

To better understand the role of FAK on the stimulation of cell migration observed in cells expressing RNAi-resistant GOLPH3 (Fig 7), we also evaluated the levels of Phospho-Y397 in cells treated with the FAK inhibitor Compound PF-562271. As expected, compared to mock-treated cells (Fig 8A, lanes 1 and 2, and Fig 8B, *Control*), incubation of control or shGOLPH3 cells with the FAK inhibitor resulted in a dramatic, significant reduction in the levels of Phospho-Y397, to 23.4 ± 3.6% and 11.0 ± 2.4%, respectively (Fig 8A, lanes 1, 2, 5 and 6, and Fig 8B, *Control* and *FAK-inh*). Of note, we found that in this condition of FAK inhibition the levels of Phospho-Y397 were also significantly different among control and shGOLPH3 cells, being also lower in shGOLPH3 cells (Fig 8A, lanes 5 and 6, and Fig 8B, *FAK-inh*), suggesting that the expression levels of GOLPH3 affected proportionally the remnant autophosphorylation activity of FAK. On the other hand, lentiviral expression of RNAi-resistant GOLPH3 in control cells incubated with the FAK inhibitor resulted in non-significant change in the levels of Phospho-Y397 compared to control cells treated with the FAK inhibitor alone (Fig 8A, lanes 5 and 7, and Fig 8B). In contrast, in shGOLPH3 cells the same combined treatment, compared to the single treatment with the FAK inhibitor, resulted in a significant increase of 50.9 ± 7.3% in the levels of Phospho-Y397 (or from 11.0 ± 2.4% to 16.6 ± 0.8% when the levels are compared to the basal levels found in control cells; Fig 8A, lanes 6 and 8, and Fig 8B), strongly indicating that the added expression of GOLPH3 stimulated the remnant autophosphorylation activity of FAK in shGOLPH3 cells. However, because this increase in the levels of Phospho-Y397 in shGOLPH3 cells was not significantly different to the levels found in control cells subjected to the same combined treatment (Fig 8A, lanes 7 and 8, and Fig 8B, *LV-GOLPH3+FAK-inh*), this result is consistent with the notion that in shGOLPH3 cells there is an available pool of FAK that could be stimulated by the levels of GOLPH3. Altogether, these results support a model in which the overexpression of GOLPH3 affects the migration of T98G cells by cooperating in the mechanism that regulates focal adhesion turnover in a FAK-dependent manner.

## Discussion

Among malignant gliomas, which are tumors that grow from glial cells, glioblastoma multiforme (GBM) is the most lethal and one of the most common primary brain tumor, characterized by rapid growth and invasion of other parts of the brain in a process that is enabled greatly by deregulation of both cell migration and extracellular matrix degradation [70]. Despite overall advances in cancer therapy, GBM remains largely resistant to treatment, with a mean survival of patients of eighteen months, time that has historically remained nearly unchanged [70, 71]. The multiforme's nature of these types of tumors is that they comprise a heterogeneous group with multiple phenotypic, genetic and epigenetic variations, highlighted by the fact that it has not been reported yet a single-mutation as a trigger of any form of GBM [71]. For this reason, it has become increasingly accepted that new therapeutic strategies for different types of GBM should consider a better understanding of the different roles that might have some molecular players [70, 71], such as GOLPH3. In our present report, we show that in T98G cells of GBM the knocking down of GOLPH3 expression, close to the levels found in non-tumorigenic astrocytes, had deleterious effects on cell migration, which correlated with a decrease in focal adhesion number and dynamics. Moreover, we found an unexpected reduction in the level of autophosphorylated FAK, suggesting that GOLPH3 is a novel FAK signaling activator. Together, our results are consistent with a role of the levels of GOLPH3 in T98G cells for the regulation of directional persistence of migration, contributing through this mechanism to their tumorigenic phenotype.

Our first observation of the effects of stably knocking down GOLPH3 in T98G cells was that it produced a dramatic change in cell morphology to a mesenchymal phenotype. This was unexpected, because several reports indicate that it is the overexpression of GOLPH3 that leads to a more mesenchymal behavior, in different tumor cell lines, including of glioblastoma and of breast cancer [10, 12, 72]. Moreover, the knocking down of GOLPH3 in the cell line MDA-MB-231 of human breast adenocarcinoma results in an apparent opposite change in cell morphology, i.e., from a mesenchymal to a more amoeboid phenotype [72]. This is important because the deregulated transition to a more mesenchymal behavior is a hallmark of cancer [73]. In fact, GOLPH3 induces epithelial-mesenchymal transition in epithelial ovarian cancer, and through the Wnt/β-catenin signaling pathway [16]. Thus, the morphology of T98G cells upon the reduction of GOLPH3 levels seemed paradoxical. However, it is also known that tumor cells can undergo transitions between mesenchymal- and amoeboid-modes of migration and invasion [74]. This suggests the possibility that in some tumor cells the levels of GOLPH3 could regulate a transition between a less active mesenchymal motility to a more amoeboid malignant behavior. This notion is supported by our subsequent findings that the knocking down of GOLPH3 affected the migration and invasion of T98G cells. These results are in agreement to the effects that similar reductions of GOLPH3 levels produce in other tumor cells, including other glioma cell lines [12], as well as in cell lines of breast cancer [72] and of hepatocellular carcinoma [75]. Furthermore, our analyses showed that even though the knocking down of GOLPH3 produced an increase in the average speed of cell motility, which is consistent with a more mesenchymal behavior [76], it also resulted in a robust decrease in directional persistence. Therefore, this behavior of T98G cells upon knocking down GOLPH3 seems to be the consequence of deregulated cell migration.

Deregulated cell migration is one of the most prominent characteristics during cancer progression, shaping the capacity of tumor cells to pathologically invade adjacent tissues [77]. It is not surprising then that regulation of cell migration is among the features attributed to the oncogenic capacity of GOLPH3 in different types of tumors, including glioblastoma [25]. For instance, it has been shown that overexpression of GOLPH3 in the human glioblastoma cell lines U87 and U251 contributes to their malignant motility by enhancing different aspects that are associated to this tumorigenic property, such as the activity of the mTOR-signaling pathway, the levels of the small GTPase RhoA, the levels of the gene expression regulator YB1, and the degradation of extracellular matrix by matrix metalloproteinase-2 (MMP-2) [12, 42]. How the levels of GOLPH3 are mechanistically related to these effects is currently unknown. Nevertheless, it is well documented the roles that RhoA and the mTOR-signaling pathway have in cell motility during oncogenic transformation. RhoA belongs to the Rho family of small GTPases, which is a ubiquitous group of proteins involved in the regulation of actin cytoskeleton dynamics [78]. Like other small GTPases, RhoA is activated by its binding to GTP, which promotes the interaction of RhoA to a variety of effector proteins, triggering several cellular responses, such as the actin cytoskeleton rearrangements that are necessaries for cell motility [78]. Thus, it has been found that abnormalities in RhoA function could lead to cancer progression through metastatic growth [79]. On the other hand, there are a number of possibilities in which the mTOR-signaling pathway participates in the regulation of malignant cell migration [80], which include the activation of RhoA [81]. How the overexpression of GOLPH3 activates RhoA and the mTOR-signaling pathway is unknown. Interestingly, in U87 and U251 cells the knocking down of GOLPH3 also decreases cell proliferation, by promoting the down-regulation of the epidermal growth factor receptor (EGFR; [59]). EGFR is a receptor tyrosine kinase that stimulates several downstream pathways that could eventually promote pro-oncogenic processes, including cell proliferation, inhibition of apoptosis, angiogenesis, and cell migration and invasion [82]. Because EGFR regulates the activities of the mTOR-signaling pathway and of RhoA [82, 83], the effects of GOLPH3 overexpression on the migration of U87 and U251 cells could be ascribed to EGFR. Thus, in T98G cells the level of expression of GOLPH3 could affect a similar control on cell motility. However, because the tumorigenic activity of EGFR in T98G cells is different to that in other GBM cell lines, including U87 cells [63], other mechanisms of GOLPH3 affecting the control of cell motility can not be ruled out. In this regard, the potential roles postulated for GOLPH3 in membrane trafficking represent other plausible possibilities for its control on malignant cell motility. In fact, in U87 and U251 cells the knocking down of GOLPH3 enhances EGFR endocytic trafficking, implying that GOLPH3 promotes the tumorigenic phenotype of these glioma cells by inhibiting the endocytosis and degradation of EGFR [59]. Likewise, the effects that the levels of GOLPH3 have on metalloproteinases in different types of tumor cell lines [12, 42, 47, 48] could be related to GOLPH3 promoting their secretory trafficking, resulting in enhanced extracellular matrix degradation. In any case, our results indicate that among the most notorious downstream effects were deregulated focal adhesion number and dynamics.

Focal adhesions are specialized, dynamic intracellular elements that are part of a larger system that allows cells to physically interact with their surrounding, including other cells and the extracellular matrix, by connecting the acting cytoskeleton with adhesion receptors at the cell surface [84]. This system is critical for cells to engage in several fundamental processes including embryonic development, tissue remodeling, and cell migration, but when it is deregulated it could lead to a variety of pathological conditions like malignant behavior [85]. Focal adhesions assembly involves the maturation of related structures called focal complexes, which are transient structures located at the periphery of the cells. Focal complexes eventually grow in size becoming functional focal adhesions that are relatively more stable. Nevertheless, focal adhesions are very dynamic, undergoing rounds of tightly regulated assembly and disassembly of many proteins [84]. Accordingly, focal adhesion dynamics could be regulated by a number of signaling pathways [86], which include those that are affected by the levels of GOLPH3. It is very likely then that the effects of GOLPH3 on cell motility in different types of tumor cells is through affecting focal adhesion dynamics. One of the best-characterized proteins recruited to focal adhesions is the cytoplasmic protein vinculin, which through its binding to actin regulates adhesions by both nucleating actin polymerization and facilitating the recruitment of actin filaments remodeling proteins [87]. Our microscopy analyses showed that the reduction of the levels of GOLPH3 resulted in a dramatic change in the localization and behavior of vinculin and mCherry-vinculin, suggesting that the effects on cell motility is by GOLPH3 affecting the signaling pathways that regulate the dynamics of focal adhesions. Importantly, at the crossroad of these signaling pathways is FAK [88]. Hence, the effects of GOLPH3 on focal adhesions could also be mediated by its role in promoting the activity of EGFR. Consistent with this possibility, it has been shown that EGFR activates FAK [89]. However, FAK is better known as being activated by integrins, which are a family of cell surface receptors that interact with the extracellular matrix eliciting signaling pathways that allow cells to change their microenvironment, including those that are critical for tumorigenic cell migration and invasion [90]. Functional integrins are heterodimers of distinct α and β subunits, and are regulated either from within the cell by binding of activators to their cytosolic domain, or from outside the cell by binding of extracellular matrix elements [91]. Activation of integrins triggers their clustering at the cell surface that eventually leads to the formation of focal adhesions [86]. Integrins are connected to the actin cytoskeleton by focal adhesions, which in fact are supramolecular structures formed by functional variations of a core multiprotein complex where FAK is early recruited and activated [92]. FAK activation at these sites involves autophosphorylation at Y397, leading to subsequent activation of several signaling pathways that include that of the non-receptor Src family of tyrosine kinases [86], that of PI3K-AKT and that of RAF-MEK-ERK [86, 93]. All those signaling pathways are upstream to that of mTOR [94], as well as to the activation of other downstream effectors that include RhoA [86]. Hence, another possibility is that in T98G cells the levels of GOLPH3 could regulate FAK-activated-dependent cell migration through integrins. In agreement with this possibility, it has been reported that the levels of GOLPH3 in HeLa cells regulate cell migration by a mechanism that involves β1 integrin [10]. Moreover, increased levels of GOLPH3 promotes *N*-sialylation of β1 integrin, as well as of other cell surface glycoproteins, leading eventually to tumorigenic signaling [10]. This notion is supported by the fact that increased, aberrant sialylation of *N*-glycans on glycoproteins, including of β1 integrin, plays important roles in cancer progression [95]. Likewise, it has been shown that sialyltransferases inhibition decreases cell migration of several cancer cell lines by decreasing the level of both sialylation of β1 integrin and activation of FAK signaling [96]. The clarification as to whether the overexpression of GOLPH3 promotes cell migration by activating FAK signaling through increased integrin sialylation, or through alternative activating mechanisms, in different types of cancer cells, including different glioblastoma cell lines, needs further investigation. However, because membrane trafficking contributes to malignant cell migration at multiple levels [97], an intriguing possibility is that GOLPH3 could be also affecting the trafficking of integrins, and thus the supply of factors important for focal adhesion dynamics. In any case, our results of increased migration and increased levels of autophosphorylated FAK in cells subjected to FAK inhibition and subsequent rescue of GOLPH3 expression, although showed to be limited in magnitude, indicate that GOLPH3 promotes FAK activity. Therefore, our data add a new piece that has to be considered for a more complete understanding of the role that GOLPH3 plays in cancer.

## Supporting information

S1 Fig The levels of GOLPH3 in wild type and shLuc T98G cells are similar. Detergent-soluble extracts of the indicated cells were prepared, and proteins were analyzed by SDS-PAGE followed by immunoblotting using antibodies to the proteins indicated on the right. The immunoblot signal of anti-β-actin was used as loading control. The position of molecular mass markers is indicated on the left. (B) Densitometry quantification of the immunoblot signal of the levels of GOLPH3 from images as shown in *A*. Bar represents the mean ± standard deviation of replicates (n = 5). *ns*, not statistically significant.
(TIFF)

S2 Fig Immunofluorescence of GOLPH3 in shLuc and shGOLPH3 cells. The indicated cells grown in glass coverslips were fixed, permeabilized, and double-labeled with rabbit polyclonal antibody to GOLPH3 and mouse mococlonal antibody to GM130. Secondary antibodies were Alexa-488-conjugated donkey anti-rabbit IgG (green channel) and Alexa-594-conjugated donkey anti-mouse IgG (red channel). Nuclei were stained with DAPI dye (blue channel). Stained cells were examined by fluorescence microscopy. Merging of the images in the green, red, and blue channels generated the third picture in each row; yellow indicates overlapping localization of the green and red channels. Bar, 10 μm. (B) Quantification of the fluorescence levels of GOLPH3 of the indicated cells from images as shown in *A*. Bar represents the mean ± standard deviation. *** *P* < 0.001.
(TIFF)

S3 Fig The levels of GOLPH3 in shGOLPH3 cells are similarly low as in astrocytes. Detergent-soluble extracts of the indicated cells were prepared, and proteins were analyzed by SDS-PAGE followed by immunoblotting using antibodies to the proteins indicated on the right. The immunoblot signal of anti-β-actin was used as loading control. The position of molecular mass markers is indicated on the left. (B) Densitometry quantification of the immunoblot signal of the levels of GOLPH3 from images as shown in *A*. Bar represents the mean ± standard deviation of replicates (n = 5). * *P* < 0.05; *** *P* < 0.001.
(TIFF)

S4 Fig Protrusions to multiple directions and of varied lengths from shGOLPH3 cells during migration. (A and B) A confluent monolayer of shGOLPH3 cells grown in a 35-mm glass-bottom culture dish was wounded with a sterile tip. The dish was transferred to a microscopy heating stage equipped with temperature, humidity and CO_2_ comptrollers, and phase-contrast images were acquired immediately, and every 5-min up to 24 h. The time after initiation of imaging is shown in the bottom left corner of each panel in hours:minutes. In *A* and *B*, filled arrows indicate the position of cells during migration, and empty arrows indicate the initial position of the cells. Bar, 20 μm.
(TIFF)

S5 Fig Example of change in directionality of an shGOLPH3 cell during migration. A confluent monolayer of shGOLPH3 cells grown in a 35-mm glass-bottom culture dish was wounded with a sterile tip. The dish was transferred to a microscopy heating stage equipped with temperature, humidity and CO_2_ comptrollers, and phase-contrast images were acquired immediately, and every 5-min up to 24 h. The time after initiation of imaging is shown in the bottom left corner of each panel in hours:minutes. The green line represents the trajectory of the position of a cell during migration obtained using the ImageJ software plug-in Manual Tracking. Bar, 50 μm.
(TIFF)

S6 Fig Example of stationary behavior of an shGOLPH3 cell during migration. A confluent monolayer of shGOLPH3 cells grown in a 35-mm glass-bottom culture dish was wounded with a sterile tip. The dish was transferred to a microscopy heating stage equipped with temperature, humidity and CO_2_ comptrollers, and phase-contrast images were acquired immediately, and every 5-min up to 24 h. The time after initiation of imaging is shown in the bottom left corner of each panel in hours:minutes. The empty arrows indicate the initial position of the cell. The green dashed-line represents the trajectory of the position of a cell during migration up to 16:20. The red dashed-line represents the trajectory at the last, stationary position during 6 h, from 16:50 to 22:50. During this period of time, the cell underwent extension and retraction of protrusions to different directions, but without net movement. Bar, 50 μm.
(TIFF)

S7 Fig Effect of nocodazole treatment in shLuc cells. The indicated cells grown in glass coverslips were left untreated (*Control*) or treated with 10 μM nocodazole (*NZ*) for 4 h at 37°C. Cells were fixed, permeabilized, and labeled with mouse monoclonal antibody to α-tubulin followed by incubation with Alexa-594-conjugated donkey anti-mouse IgG. Stained cells were examined by fluorescence microscopy. Bar, 10 μm.
(TIFF)

S1 Video Behavior of shLuc and shGOLPH3 cells during migration. (MOV)

S2 Video Behavior of mCherry-vinculin in shLuc and shGOLPH3 cells. (MOV)
