# Supplementary figures and images for: The knocking down of the oncoprotein Golgi phosphoprotein 3 in T98G cells of glioblastoma multiforme disrupts cell migration by affecting focal adhesion dynamics in a focal adhesion kinase-dependent manner
Source: PLoS One. 2019 Feb 19;14(2):e0212321. doi: 10.1371/journal.pone.0212321 (PMC6380552; doi:10.1371/journal.pone.0212321)

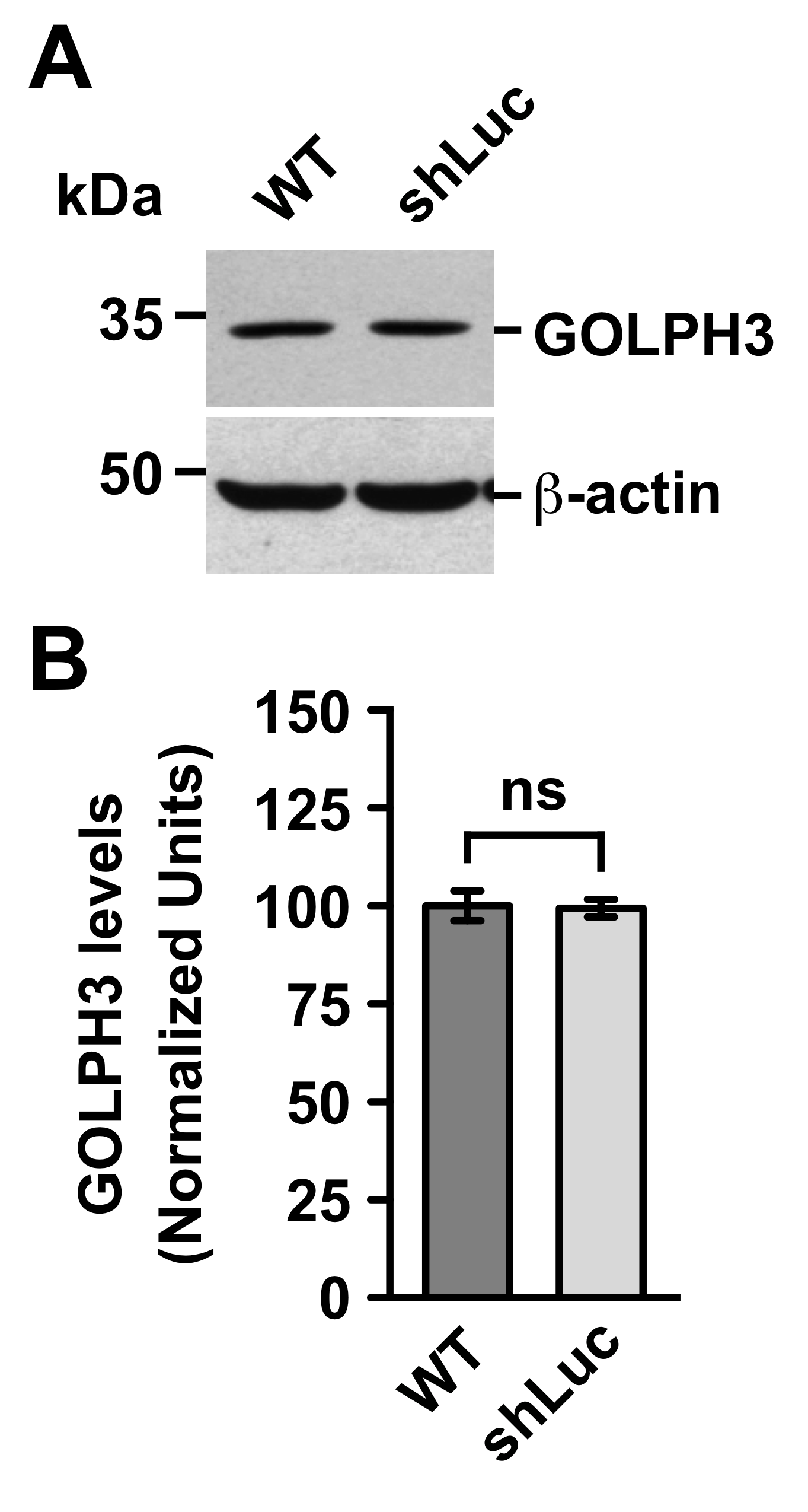

Supplement: S1 Fig — Detergent-soluble extracts of the indicated cells were prepared, and proteins were analyzed by SDS-PAGE followed by immunoblotting using antibodies to the proteins indicated on the right. The immunoblot signal of anti-β-actin was used as loading control. The position of molecular mass markers is indicated on the left. (B) Densitometry quantification of the immunoblot signal of the levels of GOLPH3 from images as shown in A. Bar represents the mean ± standard deviation of replicates (n = 5). ns, not statistically significant. (TIFF) [file pone.0212321.s001.tiff]

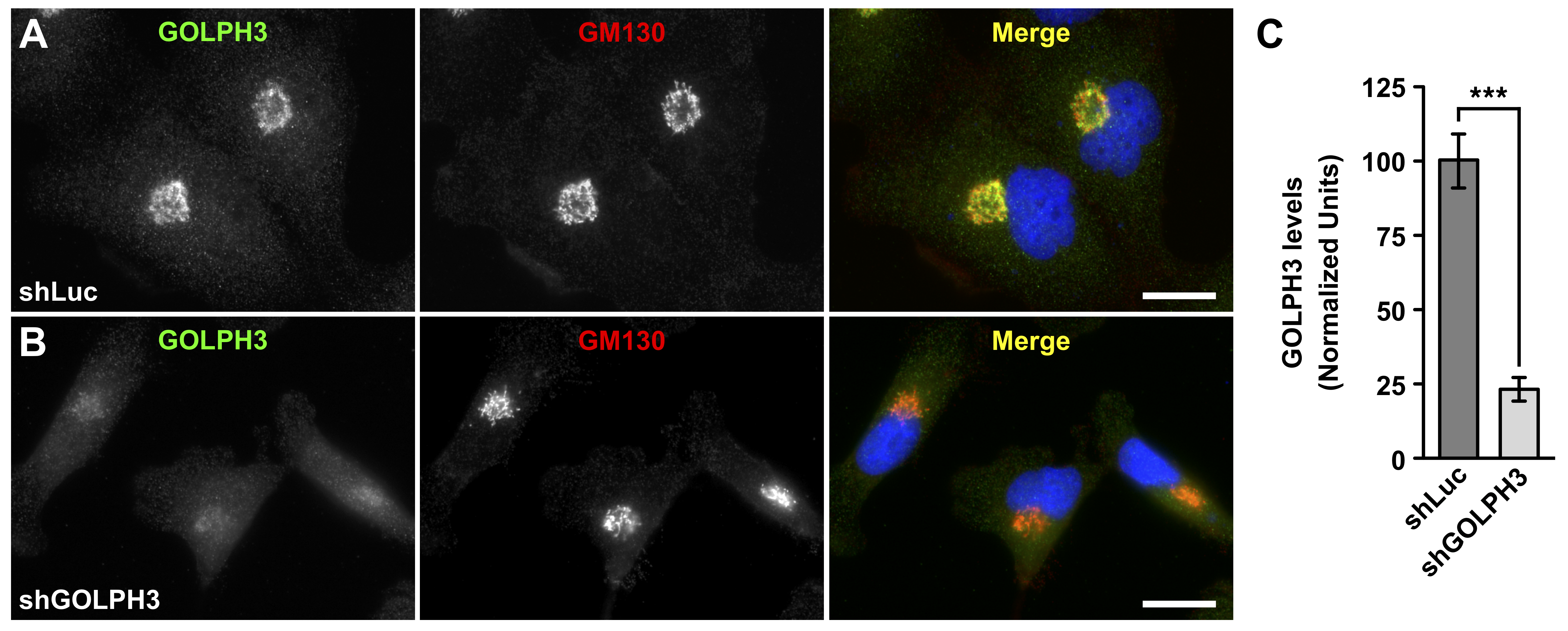

Supplement: S2 Fig — The indicated cells grown in glass coverslips were fixed, permeabilized, and double-labeled with rabbit polyclonal antibody to GOLPH3 and mouse mococlonal antibody to GM130. Secondary antibodies were Alexa-488-conjugated donkey anti-rabbit IgG (green channel) and Alexa-594-conjugated donkey anti-mouse IgG (red channel). Nuclei were stained with DAPI dye (blue channel). Stained cells were examined by fluorescence microscopy. Merging of the images in the green, red, and blue channels generated the third picture in each row; yellow indicates overlapping localization of the green and red channels. Bar, 10 μm. (B) Quantification of the fluorescence levels of GOLPH3 of the indicated cells from images as shown in A. Bar represents the mean ± standard deviation. *** P < 0.001. (TIFF) [file pone.0212321.s002.tiff]

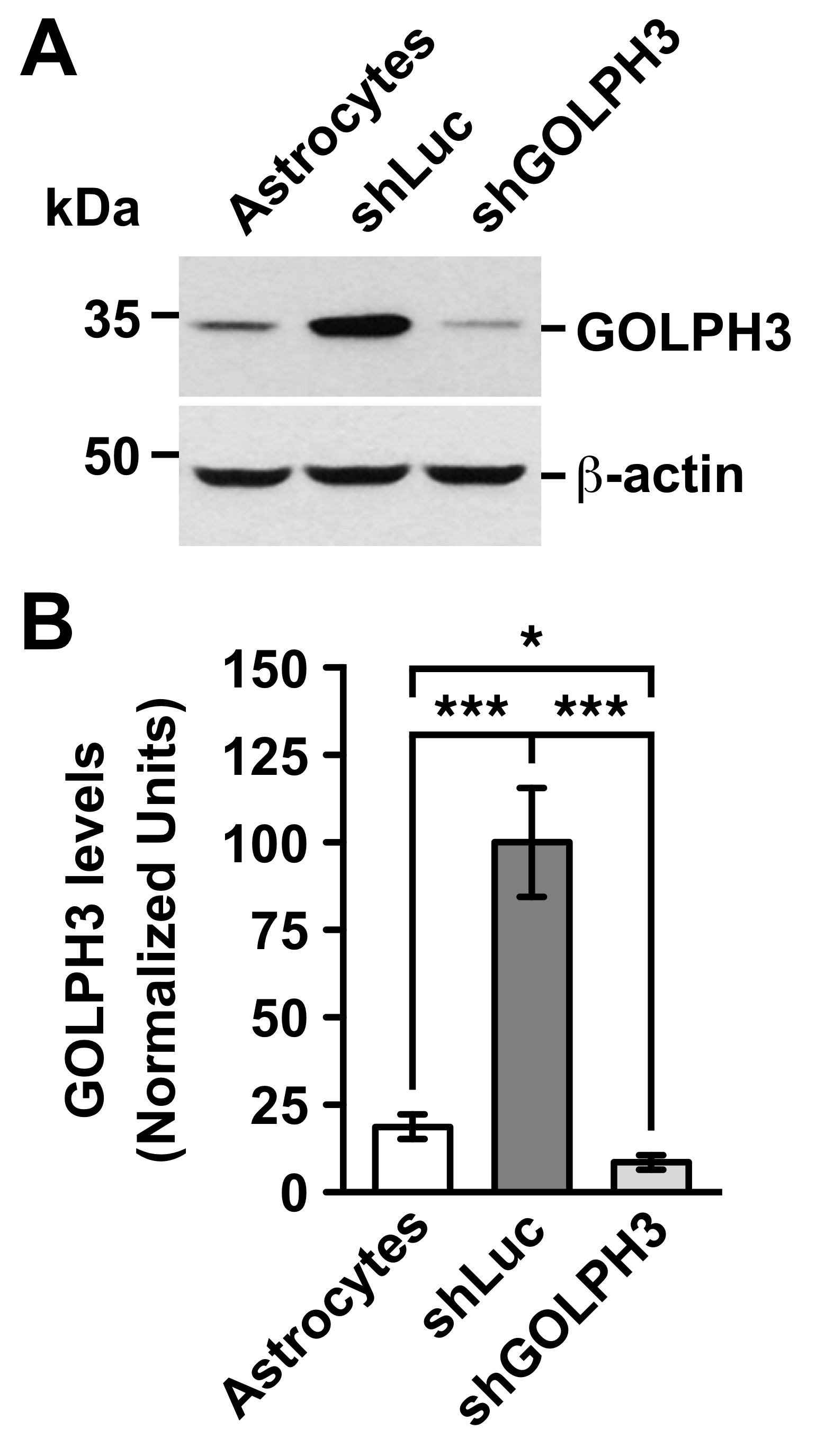

Supplement: S3 Fig — Detergent-soluble extracts of the indicated cells were prepared, and proteins were analyzed by SDS-PAGE followed by immunoblotting using antibodies to the proteins indicated on the right. The immunoblot signal of anti-β-actin was used as loading control. The position of molecular mass markers is indicated on the left. (B) Densitometry quantification of the immunoblot signal of the levels of GOLPH3 from images as shown in A. Bar represents the mean ± standard deviation of replicates (n = 5). * P < 0.05; *** P < 0.001. (TIFF) [file pone.0212321.s003.tiff]

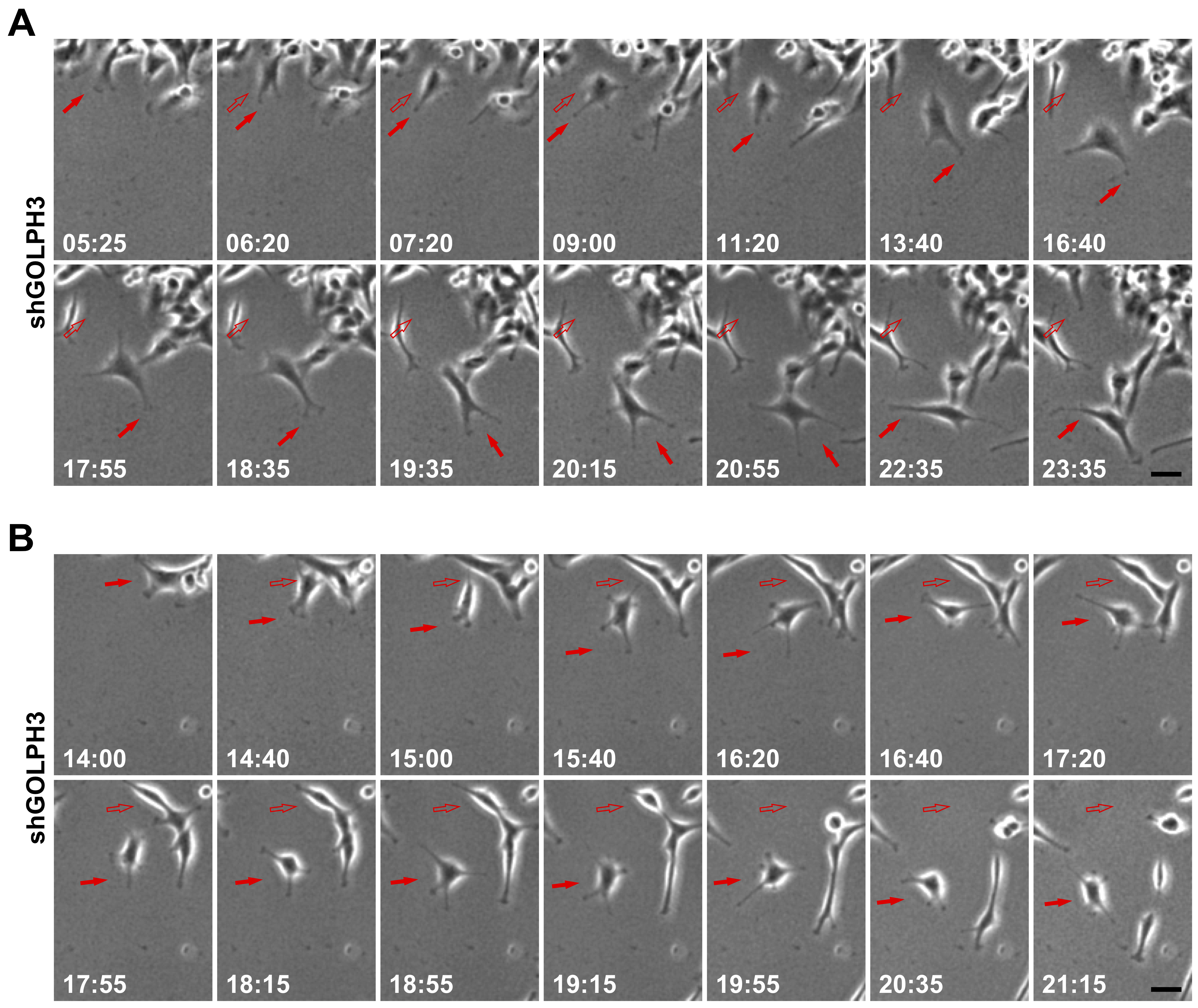

Supplement: S4 Fig — (A and B) A confluent monolayer of shGOLPH3 cells grown in a 35-mm glass-bottom culture dish was wounded with a sterile tip. The dish was transferred to a microscopy heating stage equipped with temperature, humidity and CO2 comptrollers, and phase-contrast images were acquired immediately, and every 5-min up to 24 h. The time after initiation of imaging is shown in the bottom left corner of each panel in hours:minutes. In A and B, filled arrows indicate the position of cells during migration, and empty arrows indicate the initial position of the cells. Bar, 20 μm. (TIFF) [file pone.0212321.s004.tiff]

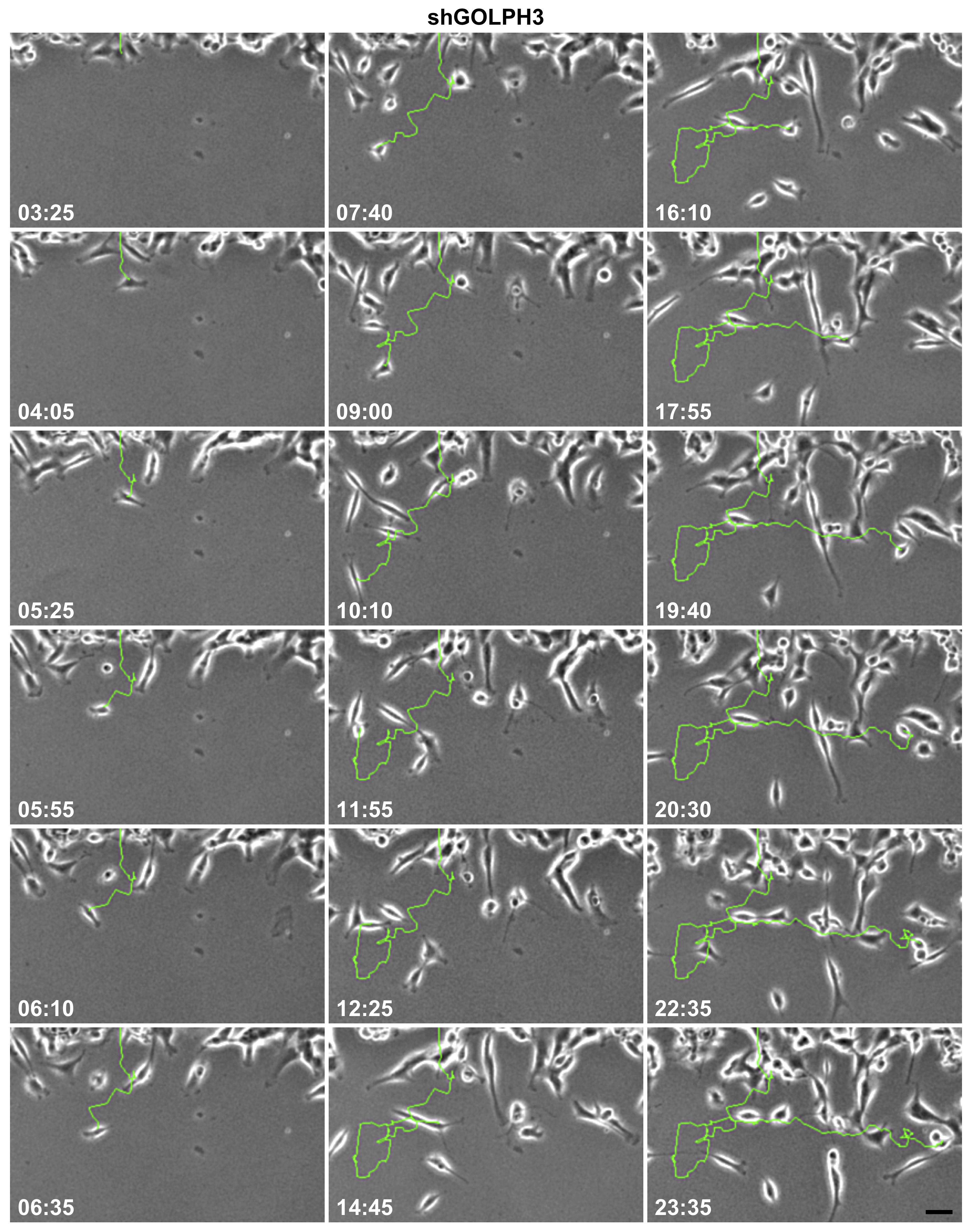

Supplement: S5 Fig — A confluent monolayer of shGOLPH3 cells grown in a 35-mm glass-bottom culture dish was wounded with a sterile tip. The dish was transferred to a microscopy heating stage equipped with temperature, humidity and CO2 comptrollers, and phase-contrast images were acquired immediately, and every 5-min up to 24 h. The time after initiation of imaging is shown in the bottom left corner of each panel in hours:minutes. The green line represents the trajectory of the position of a cell during migration obtained using the ImageJ software plug-in Manual Tracking. Bar, 50 μm. (TIFF) [file pone.0212321.s005.tiff]

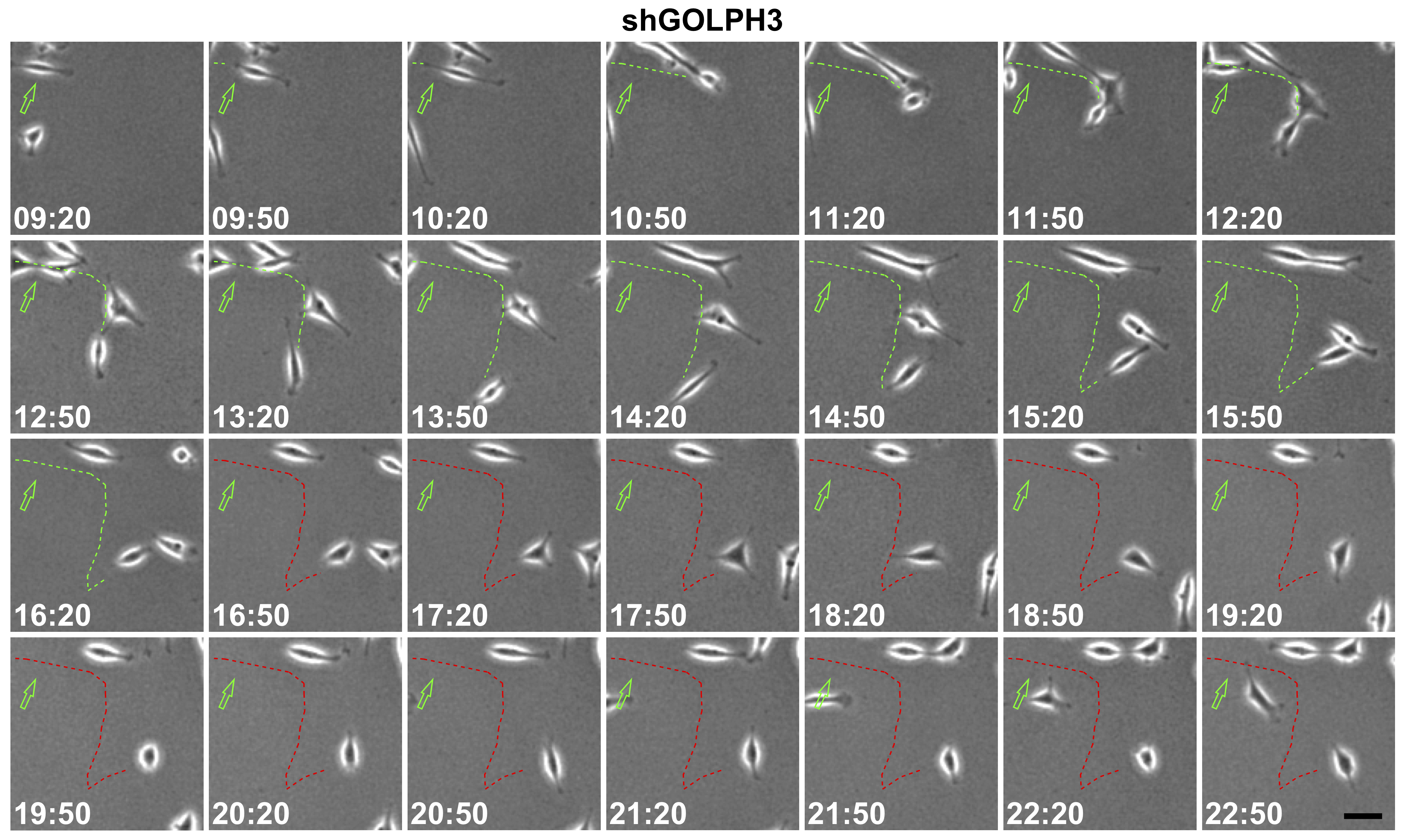

Supplement: S6 Fig — A confluent monolayer of shGOLPH3 cells grown in a 35-mm glass-bottom culture dish was wounded with a sterile tip. The dish was transferred to a microscopy heating stage equipped with temperature, humidity and CO2 comptrollers, and phase-contrast images were acquired immediately, and every 5-min up to 24 h. The time after initiation of imaging is shown in the bottom left corner of each panel in hours:minutes. The empty arrows indicate the initial position of the cell. The green dashed-line represents the trajectory of the position of a cell during migration up to 16:20. The red dashed-line represents the trajectory at the last, stationary position during 6 h, from 16:50 to 22:50. During this period of time, the cell underwent extension and retraction of protrusions to different directions, but without net movement. Bar, 50 μm. (TIFF) [file pone.0212321.s006.tiff]

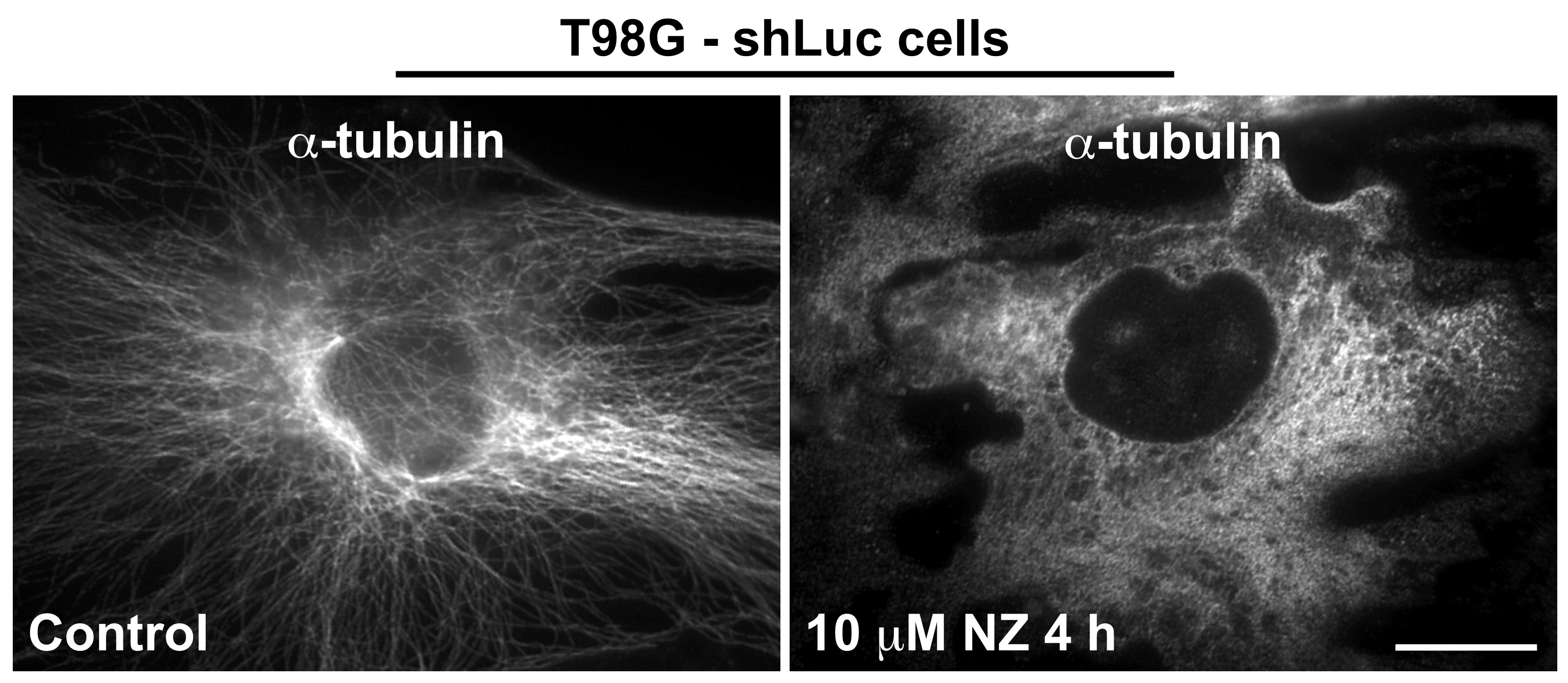

Supplement: S7 Fig — The indicated cells grown in glass coverslips were left untreated (Control) or treated with 10 μM nocodazole (NZ) for 4 h at 37°C. Cells were fixed, permeabilized, and labeled with mouse monoclonal antibody to α-tubulin followed by incubation with Alexa-594-conjugated donkey anti-mouse IgG. Stained cells were examined by fluorescence microscopy. Bar, 10 μm. (TIFF) [file pone.0212321.s007.tiff]
